# Supplementary material for: Stage-Complete Mapping of Pairwise Monocular Structure-from-Motion to Field-Programmable Gate Arrays
Source: J Imaging. 2026 Sep 18;12(9):451. doi: 10.3390/jimaging12090451 (PMC13608437; doi:10.3390/jimaging12090451)
Supplement: Supplementary file 1 [file jimaging-12-00451-s001.zip › fpga-pairwise-sfm-9ab472e/paper/Definitions/logo-mdpi-eps-converted-to.pdf]

MDPI
