# Supplementary material for: Stage-Complete Mapping of Pairwise Monocular Structure-from-Motion to Field-Programmable Gate Arrays
Source: J Imaging. 2026 Sep 18;12(9):451. doi: 10.3390/jimaging12090451 (PMC13608437; doi:10.3390/jimaging12090451)
Supplement: Supplementary file 1 [file jimaging-12-00451-s001.zip › fpga-pairwise-sfm-9ab472e/paper/main.pdf]

Article

A Stage-Complete RTL/HLS Mapping of Pairwise Monocular Structure-from-Motion to FPGA Programmable Logic

Panteleimon Stamatakis * and John Vourvoulakis

Department of Computer, Informatics & Telecommunications Engineering, International Hellenic University, Terma Magnisias, 62121 Serres, Greece; jvourv@ihu.gr

* Correspondence: pantstam@ihu.gr

Abstract: Pairwise Structure from Motion (SfM) is difficult to map to an FPGA because feature selection, correspondence search, matrix estimation, decomposition, and triangulation have different memory, arithmetic, and scheduling requirements. To the authors' knowledge, within the directly comparable FPGA literature surveyed here, this paper presents the first reported stage-complete programmable-logic mapping of pairwise monocular SfM, starting from a calibrated, pre-undistorted 1920×1080 decoded-video/RGB timing input and covering feature extraction and selection, matching, essential-matrix estimation and pruning, SVD-based pose recovery, triangulation, and source-synchronous point-coordinate output. Every listed pairwise stage is implemented in programmable logic and present in the routed design. The pipeline combines a FAST–Harris–BRIEF front end, Block-RAM-backed Top-K, spatial-bucket matching, a two-pass float32 eight-point solver, fixed-point CORDIC–Jacobi pose recovery, and fixed-point triangulation. On the VCU118/XCVU9P, Vivado 2026.1 routed all 292,748 routable nets without routing errors, met the implemented clock constraints with +0.031 ns setup slack and +0.010 ns hold slack, and generated an 80,159,324-byte bitstream. The routed design uses 12.09% of CLB LUTs, 3.49% of CLB registers, 32.94% of Block RAM tiles, and 18.90% of DSPs. Vivado Power Analyzer reports Low-confidence post-route estimates of 4.534 W under vectorless activity assumptions and 4.296 W with partial SAIF annotation. Under the stated cycle assumptions, the inferred geometry-back-end subtotal is 13.13 ms at the 1000-match ceiling. These results establish the first stage-complete, timing-closed, bitstream-generated all-PL realization of the listed pairwise SfM chain within the surveyed literature.

Keywords: FPGA; VCU118; Virtex UltraScale+; structure from motion (SfM); monocular 3D reconstruction; streaming video processing; BRIEF feature matching; eight-point algorithm; CORDIC Jacobi SVD; timing closure

Received:
Accepted:
Published:

Citation: Stamatakis, P.; Vourvoulakis, J. A Stage-Complete RTL/HLS Mapping of Pairwise Monocular Structure-from-Motion to FPGA Programmable Logic. *J. Imaging* **2026**, *1*, 0. https://doi.org/

Copyright: © 2026 by the authors. Submitted to *J. Imaging* for possible open access publication under the terms and conditions of the Creative Commons Attri- bution (CC BY) license (https://creativecommons.org/licenses/by/4.0/).

1. Introduction

Real-time three-dimensional reconstruction from monocular video is a foundational capability for autonomous robotics, augmented reality, unmanned aerial vehicles, and advanced driver assistance systems. Structure-from-Motion (SfM) recovers relative camera poses and sparse 3D scene geometry from 2D images by detecting visual features, establishing correspondences across nearby views, estimating relative camera motion, and triangulating matched points into three-dimensional space [1]. The pairwise pipeline targets calibrated, pre-undistorted monocular video with smooth bounded motion, sufficient texture and overlap, predominantly static content, a high initial inlier ratio, and non-negligible translational parallax. Section 14 states this operating domain precisely. Mature software implementations such as COLMAP [2], ORB-SLAM3 [3], and OpenCV [4,37]

provide broader estimation and optimization capabilities but execute on general-purpose CPU/GPU platforms whose latency depends on scheduling, cache behavior, memory contention, and workload.

Field-Programmable Gate Arrays (FPGAs) offer an alternative execution substrate with cycle-explicit scheduling, fine-grained parallelism, and the ability to process data in a streaming fashion without instruction-fetch and cache overhead. These properties make FPGAs attractive for embedded vision systems with strict latency and size constraints. This paper evaluates functional scheduling, numerical behavior, physical implementation, resource cost, and post-route power. Vivado Power Analyzer reports Low-confidence estimates of 4.534 W under vectorless activity assumptions and 4.296 W after applying a 70-ms partial SAIF trace, following the tool methodology in [36]; Section 10.14 gives the complete activity and confidence details.

Despite these advantages, implementing SfM on FPGAs is challenging. A complete pairwise SfM pipeline encompasses feature detection, descriptor computation, feature matching, geometric model estimation (requiring singular value decomposition), outlier rejection, pose recovery, and triangulation, each with distinct computational patterns, precision requirements, and memory access behaviors. Prior FPGA work has addressed individual stages: feature detection and description [5–12], stereo disparity computation [13, 14], and partial visual odometry front-ends [15,16]. Komorkiewicz et al. [17], the most directly comparable system among those summarized in Table 2, demonstrated SfM on a Zynq SoC at 720×576 and 50 fps; its geometric core runs in ARM software, while detection, matching, and triangulation run in programmable logic. To the authors' knowledge, the architecture reported here is the first in the directly comparable FPGA literature to implement the complete listed two-view chain in programmable logic.

The architecture presented here assigns every listed stage of its pairwise SfM chain to programmable logic. Its VCU118 wrapper accepts a calibrated, pre-undistorted 1920×1080 stream from an FMC-HDMI receiver and emits signed point-coordinate records through a source-synchronous parallel interface. The two main processing domains run at 148.5 MHz for pixels and the VHDL back end and at 49.5 MHz for the HLS solver; a separate 250 MHz board clock supports reset and receiver initialization. The 60 fps input rate is the architectural target, while the 13.13 ms value is an inferred geometry-back-end subtotal under the assumptions in Section 10, reported separately from end-to-end board throughput. The streaming front end follows the FAST-12, Harris–Stephens, and 256-bit unrotated BRIEF architecture of Stamatakis and Vourvoulakis [5]. The downstream stages are:

- **Feature selection and sorting** via a hardware tournament min-heap that retains up to 1000 of the strongest admitted features per frame and sorts them by Harris response.
- **Spatial-bucket feature matching** with a ping-pong dual-bank memory architecture whose comparison count is $O(N \cdot M)$ when the local-neighborhood occupancy M remains bounded, rather than the brute-force $O(N^2)$ count.
- **Two-pass geometric verification** using the normalized eight-point algorithm [18,19] with deterministic, pipelineable algebraic epipolar-error pruning as a throughput-oriented alternative to RANSAC [20] in the intended high-inlier regime.
- **Pose recovery** via SVD-based decomposition of the essential matrix into rotation and translation [1], with a deterministic single-correspondence cheirality selector.
- **Sparse triangulation** of up to 1000 matched points per frame pair using a pure-VHDL, DLT-derived inhomogeneous linear solver.

Floating-point arithmetic is confined to a Vitis HLS-generated eight-point solver that communicates with the VHDL pipeline through an AXI4 memory endpoint, with automatic fixed-point to IEEE 754 float32 conversion handled by a dedicated VHDL wrapper module. The implemented VCU118 design maps that endpoint to 128 KiB of on-chip Block RAM; an

external-memory deployment can replace it without changing the solver interface. The 3×3 essential-matrix SVD is implemented as a pure-VHDL CORDIC-based Jacobi eigensolver with signed 32-bit Q8.24 internal arithmetic.

Contributions

The contributions of this paper to the research community are summarized as follows.

1. **The first reported stage-complete programmable-logic mapping for pairwise SfM within the directly comparable literature surveyed here**, starting from a calibrated, pre-undistorted decoded-video/RGB timing input and covering feature extraction and selection, correspondence matching, essential-matrix estimation and pruning, pose recovery, triangulation, and source-synchronous point-coordinate output. FAST-12 detection, Harris–Stephens scoring, and the 256-bit BRIEF descriptor follow [5]; Top-K, the matcher, and the geometric back end are contributed here.
2. **A spatial-bucket matching architecture** that exploits temporal coherence to reduce the comparison count from $O(N^2)$ to $O(N \cdot M)$ for bounded local occupancy M and schedule one candidate-distance evaluation per clock after pipeline fill.
3. **A deterministic two-pass algebraic pruning scheme** optimized for the intended predominantly static, high-inlier, high-overlap selected-pair regime.
4. **A multi-precision arithmetic architecture** that uses 32-bit signed fixed-point (Q20.12, `sfixed(19, −12)`) at the streaming interfaces, an IEEE 754 float32 HLS kernel for the eight-point solver, and signed 32-bit Q8.24 internal arithmetic in the pure-VHDL CORDIC-based Jacobi eigensolver for the 3×3 SVD.
5. **A complete VCU118 implementation** that is placed, routed, timing-closed, and bitstream-generated across the 148.5- and 49.5-MHz processing domains, establishing device fit and physical timing closure for the full mapped chain.

Table 1 states the scope and provenance of every component. Reference [5] supplies the feature-front-end algorithms and its front-end-only 60 fps result. This work contributes and integrates the Top-K, matcher, complete geometric back end, full-device implementation, and scoped back-end latency analysis.

Table 1. Component scope and provenance. The front-end algorithms follow [5]; the surrounding interfaces and bounded-workload architecture are defined here.

Component	Lineage	Scope in this paper
FAST-12, Harris–Stephens, 256-bit BRIEF	Algorithmic front end based on [5]	Supplies grayscale feature locations, scores, and descriptors through the interfaces defined here.
Top-K admission, selection, and sorting	Developed for this architecture	Bounds the feature, match, solver-row, pruning, and triangulation workload.
Ping-pong spatial-bucket matcher	Developed for this architecture	Implements bounded-neighborhood descriptor matching between two internal frame slots.
Normalization, A-matrix formatting, HLS solver integration, pruning, and second solve	Developed for this architecture	Implements the two-pass essential-matrix estimation path.
Pure-VHDL SVD, single-correspondence pose selector, and triangulation	Developed for this architecture	Implements candidate pose generation, one-sample disambiguation, and point-coordinate record generation.
Inter-frame control and back-end cycle/clock methodology	Developed for this architecture	Defines pairwise scheduling and a scoped analytical timing projection distinct from measured board throughput.

The remainder of this paper is organized as follows. Section 2 provides background on the SfM problem and why it is challenging to implement in hardware. Section 3 surveys related work. Section 4 describes the overall system architecture. Sections 5–7 detail the feature selection, matching, and geometric pipeline stages. Section 8 covers accelerator integration. Section 9 describes the inter-frame pipeline scheduling strategy. Section 10 presents experimental validation and performance results. Section 11 discusses algorithmic trade-offs. Sections 12 and 13 provide discussion and future work. Section 14 states the operating domain and deployment scope, and Section 15 concludes.

2. Background: Structure from Motion

This section provides a self-contained introduction to the SfM problem for readers unfamiliar with the field. Readers experienced in computer vision may skip to Section 4.

2.1. What SfM Recovers

Structure from Motion (SfM) is the process of recovering the three-dimensional geometry of a scene and the camera's trajectory from a sequence of two-dimensional images. Given video from a single moving camera, SfM produces two outputs: (1) the *camera pose*, the rotation $\mathbf{R}$ and translation $\mathbf{t}$ describing how the camera moved between frames, and (2) a *sparse 3D point cloud* representing the visible scene structure. The reconstruction is recovered up to an unknown scale factor, as a monocular camera cannot distinguish a small nearby object from a large distant one [1].

2.2. The Five Canonical Steps

A standard SfM pipeline decomposes into five sequential stages, illustrated in Figure 1:

1. **Feature detection:** identify distinctive image locations (corners, blobs) that are likely to be recognizable across viewpoints. Common detectors include FAST [21], Harris [22], and scale-invariant methods such as SIFT [23] and ORB [24].
2. **Feature description:** encode the local image patch around each detected feature as a compact descriptor vector. Binary descriptors (e.g., ORB's 256-bit BRIEF vector [24]) enable fast matching via Hamming distance (XOR + popcount), making them especially attractive for hardware.
3. **Feature matching:** establish correspondences between features detected in two different frames. For each feature in frame N, find the most similar feature in frame $N-1$ based on descriptor distance. Brute-force matching has $O(N^2)$ complexity; spatial indexing can reduce this significantly.
4. **Geometric estimation:** from the matched point pairs, recover the camera's relative motion. Uncalibrated pixel coordinates lead to a fundamental matrix $\mathbf{F}$, whereas the calibrated K-normalized coordinates used in this design lead directly to an essential matrix $\mathbf{E}$. The eight-point method [19] estimates $\mathbf{E}$ here, followed by SVD-based decomposition to extract rotation and translation [1]. Outlier rejection (typically RANSAC [20] in software) removes erroneous matches.
5. **Triangulation:** given the recovered camera pose and matched points, compute the 3D coordinates of each point by intersecting the corresponding viewing rays from the two camera positions [1].

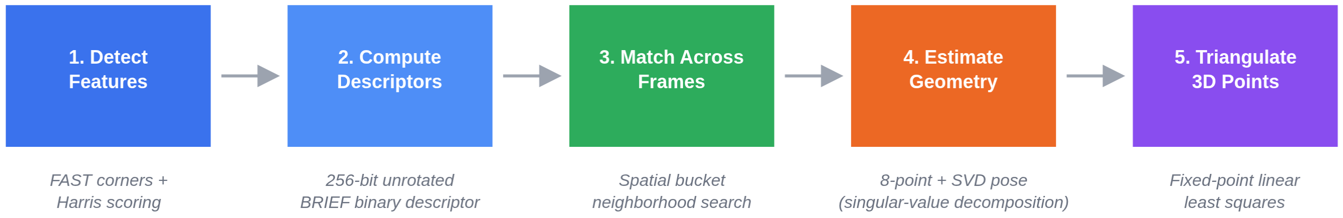

Figure 1. The five canonical Structure-from-Motion stages and the color convention used in the diagram: blue denotes feature detection and 256-bit unrotated BRIEF binary-description computation, green denotes spatial-bucket matching, orange denotes eight-point geometry and singular-value-decomposition (SVD) pose recovery, and purple denotes triangulation. Matches connect nearby selected views. An uncalibrated formulation estimates a fundamental matrix, whereas this calibrated-coordinate design estimates the essential matrix $\mathbf{E}$ and then emits scale-ambiguous 3D scene coordinates.

2.3. *Why SfM Is Hard in Hardware*

Each SfM stage presents distinct implementation challenges for FPGA hardware:

- **Feature detection** is naturally suited to streaming hardware: pixel data arrives in raster order, and local operations (convolutions, threshold comparisons) map directly to shift-register pipelines. This stage is the best-understood in the FPGA literature [5–12].
- **Feature matching** requires random-access memory patterns (comparing a query feature against a database of candidates) and large descriptor storage (1000 features $\times$ 256 bits $=$ 256 Kb). Brute-force matching's $O(N^2)$ complexity is prohibitive; spatial indexing structures must be designed for the BRAM access patterns available in FPGA fabric.
- **Geometric estimation** involves solving large linear systems (the eight-point algorithm produces a 1000×9 matrix), computing matrix decompositions (SVD), and performing iterative numerical algorithms (inverse power iteration, Jacobi rotations). These operations require either floating-point arithmetic or carefully managed fixed-point precision, and their irregular control flow is poorly suited to streaming architectures.

- **Outlier rejection** in software commonly uses RANSAC, a randomized iterative algorithm requiring many model evaluations. Although a fixed iteration cap can make RANSAC's latency deterministic, repeatedly evaluating hypotheses is a poor fit for this design's frame-time and resource budget.
- **Triangulation** requires solving small (4×4) linear systems for each matched point pair. While each individual solve is straightforward, processing 1000 points sequentially at real-time rates requires careful pipeline scheduling.

2.4. Why FPGAs

FPGAs offer several properties that make them attractive for embedded SfM despite the implementation challenges above:

- **Cycle-explicit control:** custom RTL stages have explicit state transitions and bounded local loops under fixed inputs and no back-pressure. System-level latency can nevertheless vary with candidate occupancy, AXI handshakes, CDC FIFOs, arbitration, and external memory. A hard real-time guarantee therefore requires full-device integration and a complete transaction-level bound.
- **Streaming parallelism:** active pixels can be processed as they arrive, one accepted pixel per clock. A 1920×1080 active image contains 2,073,600 active-pixel cycles; the complete 1080p60 raster, including blanking, contains 2,475,000 link-clock cycles.
- **Application-specific specialization:** streaming dataflow and custom arithmetic precision avoid instruction, cache, and general-purpose compute overhead. The two Low-confidence post-route estimates reported below quantify routed-device power under vectorless and partial-SAIF tool models; representative workload activity and physical rail measurements provide complementary deployment evidence.
- **Custom precision:** unlike CPUs locked into standard floating-point widths, FPGAs can implement application-selected word lengths. This design uses 32-bit Q20.12 interfaces after coordinate normalization and signed 32-bit Q8.24 arithmetic inside the SVD and triangulation solvers. These formats bound hardware cost, while Section 10.5 characterizes their finite-precision operating envelope.

3. Related Work

The following review separates direct pairwise-geometric precedents from complementary feature-front-end and SLAM accelerators. Feature accelerators establish relevant streaming and descriptor architectures, while complete SLAM systems provide processor-partitioning and optimization context; neither category is presented as a direct implementation of the complete pairwise geometric back-end proposed here.

3.1. FPGA Feature Extraction

Representative FPGA implementations of visual feature detectors and descriptors include the following; Reference [5] provides a broader survey. Weberruss et al. [6] demonstrated ORB feature extraction and matching in hardware, later extending to multilevel ORB with pyramid processing on FPGA [7]. Fang et al. [8] implemented an FPGA-based ORB extractor targeting real-time visual SLAM. Wasala et al. [9] achieved real-time ORB extraction for UHD video streams on an embedded FPGA platform. Belmessaoud et al. [10] presented a complete FPGA implementation of ORB detection and matching. Huang and Zhang [11] proposed a high-efficiency FPGA-based ORB matching system. Fularz et al. [12] implemented FAST detection and BRIEF descriptor matching in hardware, achieving high throughput. Huang et al. [25] presented an FPGA architecture combining FAST and BRIEF for on-board corner detection and matching.

Stamatakis and Vourvoulakis [5] implemented a FAST–Harris–BRIEF front end (FAST-12 detection with a 16-pixel Bresenham circle, Harris–Stephens corner-response scoring, and 256-bit unrotated BRIEF descriptors from 27×27 patches) for 1920×1080 video at 60 fps. The architecture below integrates that algorithmic front end with the complete pairwise back end and reports full-device implementation evidence for the combined design.

3.2. FPGA Visual SLAM and Visual Odometry

Several works have addressed hardware acceleration of visual SLAM subsystems. Liu et al. [15] proposed eSLAM, an energy-efficient accelerator for ORB-SLAM on FPGA that achieves significant speedups over ARM processors while reducing energy consumption. Wang et al. [16] presented ac2SLAM, an FPGA-accelerated SLAM system incorporating heapsort-based feature selection and parallel keypoint extraction. Fularz et al. [26] demonstrated an FPGA implementation of the essential matrix estimation using RANSAC with both the 8-point and 5-point methods, representing one of the few hardware implementations of geometric verification.

The most closely related work is by Komorkiewicz et al. [17], who presented a heterogeneous Zynq SoC system for real-time SfM at 720×576 at 50 fps targeting automotive ADAS applications. Their system implements Harris corner detection, SAD-based block matching, and DLT triangulation in FPGA fabric, while the ARM Cortex-A9 processor running Linux performs the geometric core: RANSAC-based fundamental-matrix estimation (including SVD), projection-matrix recovery, and associated linear algebra. The reported processor path handles approximately 400 matched points with up to 10 RANSAC iterations per frame within a 15 ms budget. In contrast, the architecture reported here maps coordinate normalization, the HLS-generated eight-point solver, algebraic pruning, essential-matrix extraction, fixed-point SVD-based pose recovery, and triangulation to programmable logic, and the complete VCU118 implementation is routed, timing-clean, and bitstream-generating.

Concurrent and recent work has begun to accelerate the SLAM optimization back-end on FPGA. El Bouazzaoui et al. [27] presented an HLS/OpenCL implementation of the HOOFR-SLAM front-end on a CPU–FPGA architecture, with feature extraction and matching on FPGA but pose estimation and loop closure on the embedded CPU. Arora et al. [28] (SoC-SLAM, 2025) implemented a bundle-adjustment accelerator (BAX) on a Zynq-class device using a hardware/software co-design approach, completing one BA pass in ∼63 ms on FPGA while keeping other stages in software. Kim et al. [29] (SuperNoVA, ASPLOS 2025) presented an algorithm-hardware co-design that accelerates pose-graph optimization for resource-constrained SLAM on a custom hardware substrate. These works target iterative non-linear optimization stages such as bundle adjustment and pose-graph optimization. They complement the all-PL selected-pair geometry pipeline studied here and can consume its relative-pose and sparse point records in future multi-frame systems. None of these systems implements the same pairwise geometric back end entirely in programmable logic.

3.3. FPGA Stereo Vision

FPGA implementations of stereo matching are well-established. Mattoccia [13] surveyed stereo vision algorithms suitable for FPGA implementation. Zhao et al. [14] presented FP-Stereo, a hardware-efficient stereo vision system for embedded applications. While stereo vision produces dense depth maps from rectified image pairs with known baseline, this architecture targets the fundamentally different problem of monocular SfM: recovering camera motion and sparse 3D point records from a single moving camera without a known baseline, up to an unknown scale factor.

3.4. FPGA Matrix Decomposition

Singular Value Decomposition (SVD) is central to many computer vision algorithms. Wang and Zambreno [30] implemented the Hestenes–Jacobi algorithm for SVD on FPGA, demonstrating that iterative rotation-based SVD can be expressed in hardware. Our design employs a pure-VHDL CORDIC-based Jacobi eigensolver with signed 32-bit Q8.24 internal arithmetic for the 3×3 essential-matrix SVD, requiring no floating-point hardware, and inverse power iteration with Cholesky factorization for the larger eight-point system, implemented as a Vitis HLS-generated float32 kernel.

3.5. Software SfM Systems

COLMAP [2] implements an incremental SfM pipeline with bundle adjustment. ORB-SLAM3 [3] extends the ORB-SLAM framework [31] with visual–inertial and multi-map capabilities. OpenCV [4,37] provides reference implementations of standard SfM building blocks. These software systems serve as algorithmic references for the hardware pipeline, although their execution time depends on the host platform and workload.

3.6. Summary of the Gap

Table 2 summarizes the stage coverage reported by the directly comparable FPGA vision works reviewed here. To the authors' knowledge, the present design is the first reported architecture in this literature to assign programmable logic to every listed two-view stage. The input boundary is an already decoded, calibrated, and pre-undistorted RGB/timing bus; HDMI/TMDS decoding and lens undistortion define the upstream interface boundary.

Table 2. Comparison of reported FPGA vision implementations by pipeline-stage coverage. PL means that the cited work's primary architecture reports the stage in programmable logic (FPGA fabric or HLS-synthesized RTL), SW means embedded-processor software, and N/A means not implemented in the cited scope. The labels compare stage allocation.

Work	Detection	Desc.	Matching	Geom. Verif.	Pose Est.	Triang.
Weberruss et al. [6,7]	PL	PL	PL	N/A	N/A	N/A
Fang et al. [8]	PL	PL	N/A	N/A	N/A	N/A
Wasala et al. [9]	PL	PL	N/A	N/A	N/A	N/A
Belmessaoud et al. [10]	PL	PL	PL	N/A	N/A	N/A
Fularz et al. [12]	PL	PL	PL	N/A	N/A	N/A
Fularz et al. [26]	N/A	N/A	N/A	PL+SW	PL+SW	N/A
Liu et al. / eSLAM [15]	PL	PL	PL	N/A	N/A	N/A
Wang et al. / ac2SLAM [16]	PL	PL	N/A	N/A	N/A	N/A
Komorkiewicz et al. [17]	PL	N/A	PL	SW	SW	PL
Stamatakis and Vourvoulakis [5]	PL	PL	N/A	N/A	N/A	N/A
This work	PL	PL	PL	PL	PL	PL

4. System Architecture

4.1. Platform and Data Flow

The implemented platform is the AMD/Xilinx VCU118 evaluation board with the `xcvu9p-flga2104-2L-e` Virtex UltraScale+ FPGA [34]. Its wrapper is pin-mapped to a Digilent FMC-HDMI receiver on J2/HPC1 for an ADV7611-decoded 24-bit RGB bus, horizontal and vertical synchronization, data enable, and a 148.5 MHz link clock [35]. All receiver data, timing, I^2C, reset, and interrupt pins use LVCMOS18. The wrapper phase-shifts the link clock for input capture and transfers the complete video word into the raw-link-clock domain. A 250 MHz differential board clock supports reset and receiver initialization. The ADV7611 is configured over I^2C; the processing core remains in reset

until initialization completes, link presence is reported, and the capture MMCM locks. Link presence does not itself verify the expected 2200×1125 total raster or 1080p60 timing. Separately, the FMC VADJ rail must be set physically to 1.8 V before use; the bitstream cannot program or verify that rail.

The stage-complete programmable-logic design contains the streaming feature front end, Block-RAM-backed Top-K, the matcher, the geometry pipeline, the 49.5 MHz HLS solver, AXI clock conversion/interconnect, and a 128 KiB on-chip BRAM endpoint. The BRAM endpoint supplies the solver's memory transactions without requiring an embedded processor or external DDR. Each valid point-coordinate record is emitted on J22/HSPC as `record[107:0]` = {write-enable, address[10:0], X[31:0], Y[31:0], Z[31:0]}; X, Y, and Z are signed Q8.24 words. The 108 record bits use LVCMOS18 and are sampled on the rising edge of a forwarded LVDS clock. Post-route output timing closes against the stated 1.0 ns setup and 0.5 ns hold interface constraints.

Vivado 2026.1 completed placement and routing of this board design. All 292,748 routable nets are fully routed with zero routing errors. Static timing analysis reports +0.031 ns worst setup slack, +0.010 ns worst hold slack, and +1.347 ns worst pulse-width slack, with zero failing endpoints. The resulting bitstream is 80,159,324 bytes. These results establish complete physical implementation, timing closure under the stated constraints, and bitstream generation.

The smaller deployment candidate is `xczu19eg-ffvc1760-2-e`. Logic optimization of the full register-backed design required 532,817 of its 522,720 CLB LUTs, a measured excess of 10,097 LUTs (1.93%, or about 2%). This narrow gap identifies the remaining small-device optimization target. The Block-RAM-backed Top-K organization proven in the complete VCU118 implementation provides the direct architectural route for a future exact-part XCZU19EG build.

Table 3 summarizes the available simulation and physical-implementation evidence.

Table 3. Implementation status by evidence stage. PL denotes programmable logic and OOC denotes out-of-context synthesis.

Stage	Status	Evidence and claim boundary
Focused RTL simulation	Completed	Top-K cadence and output equivalence were verified. The fixed-point SVD passes all 72 scored nondegenerate temporal-envelope cases and all 36 planar stress cases, while deliberately ill-conditioned cases define the tested numerical boundary. Triangulation is covered by controlled numerical, implemented-schedule, and output-equivalence tests.
VCU118 board-interface simulation	Completed	The focused wrapper test covers 45/50/55% link-clock duty points, 27-bit RGB/timing alignment, reset release, no dropped eligible samples, point-record packing, and forwarded-clock sampling. Its scope is digital wrapper behavior; physical card, cable, and receiver characterization belongs to live-board deployment.
Final VCU118 parent synthesis	Completed	The board wrapper and all OOC partitions were linked for `xcvu9p-flga2104-2L-e`; the implemented design contains no black boxes.
VCU118 placement and physical optimization	Completed	Board pins, input/output timing, generated clocks, and path-specific internal timing schedules were applied before placement and physical optimization.
VCU118 routing and timing	Completed; timing met	292,748/292,748 routable nets are fully routed, with no routing errors or Error/Fatal DRC violations. WNS is +0.031 ns and WHS is +0.010 ns.
VCU118 bitstream	Generated	The bitstream is 80,159,324 bytes with SHA-256 `1257def2115839cee1ea60 30b8d4954e6655baa3d3849de3f6818bcb2a208e13`.
VCU118 live-board evaluation	Planned deployment step	Live HDMI capture, point-record reception, sustained-throughput measurement, and physical rail-power measurement extend the completed board-level implementation evidence.
XCZU19EG design-space mapping	1.93% LUT overage in the studied configuration	The full register-backed design reached 532,817/522,720 CLB LUTs (101.93%), motivating direct implementation of the Block-RAM-backed organization on this smaller device.

The design distributes the 148.5 MHz HDMI link clock to the streaming front end, wrapper controller, and pure-VHDL geometry stages. Arithmetic paths whose registers are intentionally separated by multi-cycle schedules use source–destination-specific setup/hold exceptions matched to their RTL update intervals. The triangulator's feedback path is held for 12 clocks in RTL and constrained for 12-cycle setup/11-cycle hold analysis. Pose, SVD, and pruning use their separately verified path schedules. The HLS-generated eight-point solver instead runs on a physical 49.5 MHz clock derived by divide-by-3, and AXI clock-converter IP bridges the two domains. Final post-route analysis covers every declared primary and generated clock, including the 250 MHz board clock, 148.5 MHz link/capture clocks, 49.5 MHz solver clock, and forwarded point clock.

The primary data flow begins after lens undistortion and calibration:

$$\text{Decoded, Pre-Undistorted Video} \rightarrow \text{Feature Extraction} \rightarrow \text{Matching} \\ \rightarrow \text{Geometry Pipeline} \rightarrow \textbf{Sparse 3D Point-Record Stream}$$

The pipeline consumes the decoded video/timing bus and emits point-coordinate records in signed Q8.24 form. Debug-video signals are retained within the wrapper and reduced onto one status LED for observability. The board wrapper uses the source-synchronous point-record interface described above as its physical output.

Figure 2 separates the board interfaces, the 148.5 MHz streaming and geometry logic, and the 49.5 MHz HLS solver.

The `processing_module` contains all custom logic. Internally, it decomposes into a streaming pixel-processing front end and a decoupled post-frame geometry back end. Most

custom geometry logic remains in the 148.5 MHz pixel-clock domain; only the HLS solver uses the divided 49.5 MHz clock:

```
processing_module
 |-- [1] rgb2gray          (streaming, 1 pixel/clock)
 |-- [2] feature_frontend  (streaming)
 |    `-- FAST + Harris + unrotated BRIEF + Top-K
 |-- [3] feature_matcher   (ping-pong banks, spatial buckets)
 `-- [4-9] system_controller (geometry back-end)
     |-- [4] coordinate_normalizer
     |-- [5] eight_point_formatter
     |-- [6] solver_wrapper (pix_clk 148.5 MHz)
     |    `-- AXI clock conversion
     |        + HLS kernel (kernel_clk 49.5 MHz)
     |        + 128 KiB BRAM endpoint
     |-- [7] algebraic_pruner
     |-- [8] pose_recovery
     |    `-- svd_3x3 (pure VHDL, CORDIC Jacobi)
     `-- [9] triangulator (pure VHDL linear solver)
          `-- 108-bit point-record output + forwarded clock
```

Stages [1]–[2] implement the feature-front-end algorithms described in [5] and connect them to the interfaces and bounded-workload architecture defined here. The grayscale conversion is the fixed integer expression $(76R + 151G + 28B)/256$. A rolling 27×27 window supplies a radius-3, 16-pixel FAST circle; a candidate requires 12 contiguous circle samples to be brighter or darker than the center by the configured threshold of one grayscale level. Prewitt gradients and a 3×3 Gaussian smoothing kernel feed the Harris–Stephens response $\det(M) - 0.05\,\mathrm{trace}(M)^2$, with an admission threshold of 10,000. The same window produces an unrotated 256-bit BRIEF descriptor from fixed pixel-pair comparisons. A valid record comprises the aligned row, column, Harris score, and descriptor and is offered to the Top-K pool. Top-K in stage [2] and stages [3]–[9] define the bounded-workload and geometry architecture summarized in Table 1.

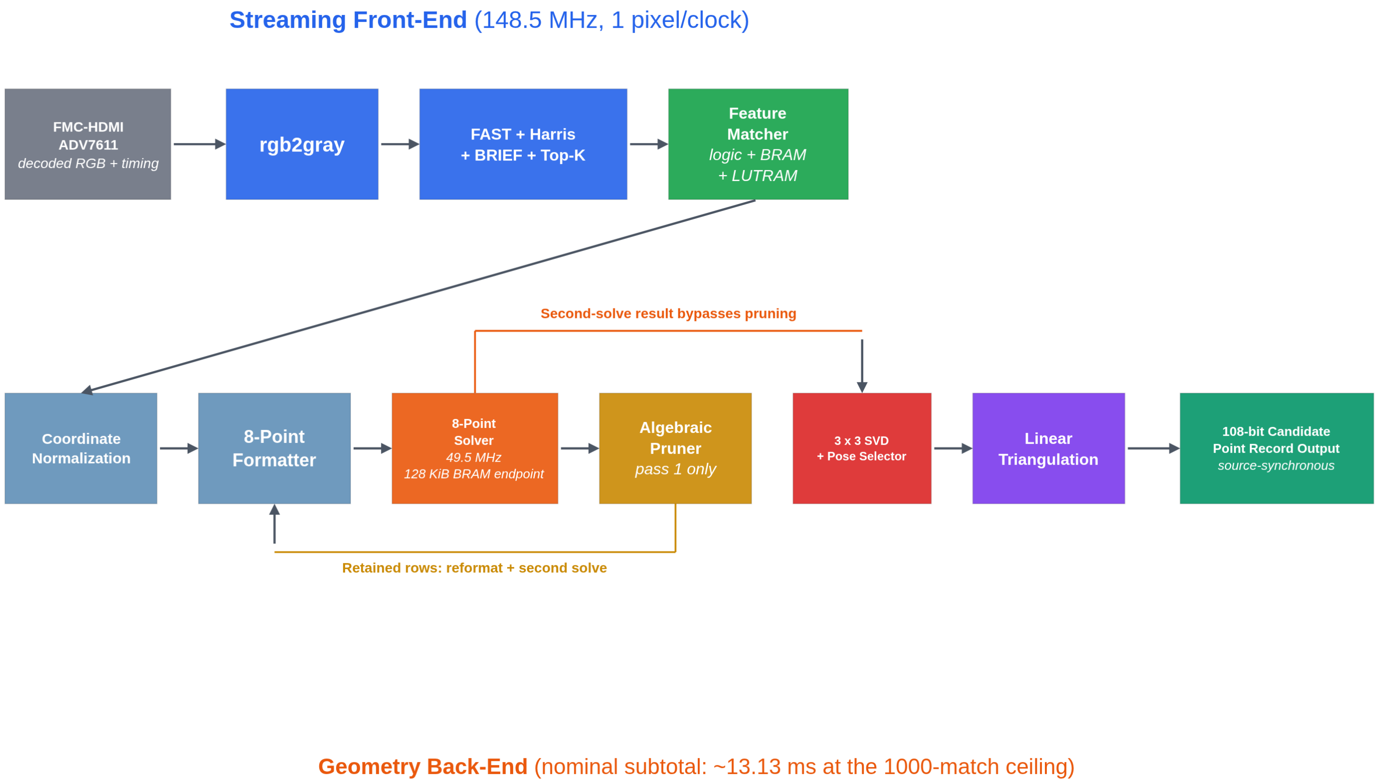

Figure 2. Implemented VCU118 architecture and color mapping. Gray denotes the board interfaces: a Digilent FMC-HDMI/ADV7611 decoded input on J2/HPC1 and a source-synchronous 108-bit point-record output on J22/HSPC. Blue denotes the 148.5 MHz streaming feature front end and Block-RAM-backed Top-K; green denotes matching; orange denotes the 49.5 MHz HLS solver and its on-chip BRAM transaction endpoint; yellow denotes pruning; red denotes pose recovery and SVD; and purple denotes triangulation. Lens undistortion is outside the design. The 13.13 ms value is a nominal geometry-back-end subtotal, not measured board latency.

4.2. Fixed-Point Arithmetic Design

The streaming interfaces and most pixel-domain computations use a 32-bit signed fixed-point format, `sfixed(19 downto -12)`, denoted Q20.12 because it provides 20 bits at and above the binary point (including sign) and 12 fractional bits:

- **Range:** $[-524288, +524287.999756]$
- **Resolution:** $2^{-12} = 0.000244$

This format is stored as a 32-bit `std_logic_vector`. The shared-package helper `to_fx()` maps SLV to sfixed, while `to_elem()` maps sfixed to SLV. Arithmetic-heavy elimination and SVD stages use signed 32-bit Q8.24 internally and explicitly realign values at their Q20.12 boundaries. These representation choices bound word length; Section 10.5 characterizes the resulting numerical range and precision, including overflow-sensitive and weak-geometry cases.

A critical exception arises for reciprocal focal lengths. For a camera with focal length $f_x = 1735.037$ pixels, the reciprocal $1/f_x = 0.000577$ requires more than 12 fractional bits to represent accurately. In the standard `sfixed(19,-12)` format, this value quantizes to 0.000488, a 15.4% error that propagates through all downstream geometry. To address this, reciprocal focal lengths use `sfixed(3 downto -28)` with a 2^{-28} representation step before the result is resized at the downstream interface.

4.3. Data Storage Architecture

Understanding where data resides is essential for reasoning about the pipeline's timing, resource cost, and throughput. Table 4 summarizes the storage taxonomy used throughout the design.

Table 4. Storage types used across the pipeline and their role in the design.

Storage Type	Characteristics	Usage in Pipeline
Block RAM (BRAM)	Dedicated 36 Kb dual-port memory; synchronous read.	Row buffers, the packed 1024×349 Top-K pool, two CPRAM feature banks, match lists, the formatted A matrix, and the 128 KiB solver-transaction endpoint.
Distributed RAM (LUTRAM)	LUT-based memory used when an access pattern does not map efficiently to a block-RAM primitive.	CDRAM bucket directories and small local lookup structures.
Flip-Flops (FFs)	Individual storage elements; combinational (zero-latency) read.	Pipeline registers (P1–P6 in pruner), FSM state, counters, accumulators, the Hamming-distance pipeline, and individual matrix elements during computation.
AXI memory endpoint	Burst-addressed memory visible to the wrapper and HLS master.	The VCU118 proof uses 128 KiB of on-chip BRAM for solver transactions. An external-DDR deployment may replace this endpoint, but its service latency is not measured here.
DSP Slices	Dedicated multiply-accumulate blocks with internal pipeline registers.	Harris response computation (I_x^2, I_y^2, $I_x I_y$), sfixed multiplication in coordinate normalizer.

4.4. Design Parameters

Table 5 consolidates the principal design parameters fixed at synthesis time for the reported operating point.

Table 5. Principal design parameters of the pipeline.

Category	Parameter	Value	VHDL Module
Video I/O	Input resolution	1920×1080	Testbench timing
	Target frame rate	60 fps	FMC-HDMI receiver operating point [35]
	Pixel clock	148.5 MHz	FMC-HDMI receiver operating point [35]
Feature Detection	FAST-12 threshold	1	`prewitt.vhd`
	Harris score threshold	10,000	`prewitt.vhd`
	Descriptor length	256 bits (BRIEF)	`prewitt.vhd`
Feature Selection	Top-K capacity	1000 features	`topk_tournament_min_tree.vhd`
Feature Matching	Bucket size	32×32 pixels (shift = 5)	`feature_matcher.vhd`
	Max features per bucket	15	`feature_matcher.vhd`
	Search neighborhood	3×3 buckets ($<$ 64 px/axis; $\leq$ 89.1 px Euclidean)	`feature_matcher.vhd`
	Hamming acceptance threshold	$\leq$ 106 / 256 bits	`feature_matcher.vhd`
	Displacement indicator	60 pixels on an accepted match; not the sole bank-advance gate	`feature_matcher.vhd`
	Active candidate streams	1	`feature_matcher.vhd`
Arithmetic	Primary interface fixed-point format	signed Q20.12, `sfixed(19,-12)` = 32 bits	`sfm_types_pkg.vhd`
	Reciprocal precision format	`sfixed(3,-28)` = 32 bits	`coordinate_normalizer.vhd`
Camera Intrinsics	Focal lengths (f_x, f_y)	1735.037, 1736.430 px	`coordinate_normalizer.vhd`
	Principal point (c_x, c_y)	956.295, 541.765 px	`coordinate_normalizer.vhd`
Geometric Pipeline	Epipolar pruning threshold	0.10 (normalized coords)	`algebraic_pruner.vhd`
	A matrix capacity	1000 rows × 9 cols × 32 bits	`sfm_controller.vhd`
	Solver algorithm	8-point (Hartley normalized)	HLS kernel (float32)
	SVD algorithm	CORDIC-based Jacobi eigensolver (3×3)	Pure VHDL (signed 32-bit Q8.24 internal)
	Triangulator feedback wait	12 pixel clocks	`trig_dlt_solver.vhd`
	Max emitted points	1000	`triangulator.vhd`
Implementation Target	Board / FPGA	VCU118 / `xcvu9p-flga2104-2L-e`	N/A
	Available CLB LUTs	1,182,240	N/A
	Available CLB registers	2,364,480	N/A
	Available BRAM36-equivalent tiles	2160	N/A
	Available UltraRAM (288 Kb)	960	N/A
	Available DSPs	6840	N/A

Note on FAST threshold. The FAST-12 threshold of 1 represents the raw pixel intensity difference used in the initial corner detection pass (i.e., a pixel is a FAST candidate if at least 12 contiguous pixels on the Bresenham circle differ from the center pixel by more than 1 intensity level). This deliberately permissive threshold favors candidate generation before the subsequent Harris response gate. Only candidates whose Harris score exceeds 10,000 are presented to the Top-K selection stage.

5. Feature Selection and Sorting

5.1. Algorithmic Role

The FAST-12 detector with Harris scoring can fire thousands of candidate keypoints per 1920×1080 frame, with the exact count governed by scene texture density (blank walls yield very few; cluttered indoor scenes yield many). Processing an unbounded candidate set through matching and geometry would be prohibitively expensive in hardware. The

compile-time value $K = 1000$ is a deterministic storage and execution-time ceiling chosen to bound the feature pool, match list, nine-column A matrix, pruning pass, and triangulation loop; it is not claimed to be an empirically optimal feature count. The Top-K stage retains at most 1000 admitted features according to Harris response, and all downstream capacities use the same ceiling.

5.2. Tournament Min-Heap Architecture

Feature selection is implemented as a hardware tournament min-heap of logical capacity $K = 1000$. The packed pool has 1024 physical entries so that the 349-bit feature records map regularly into Block RAM. The min-heap uses zero-based indices and maintains the invariant that the root (index 0) is the *smallest* Harris score in the pool. As candidate features arrive from the detector:

1. **If the pool is not full** ($n < K$): insert the candidate and bubble up to restore the heap property. Cost: $O(\log K) \approx 10$ indexed read/write steps in the functional schedule.
2. **If the pool is full and the candidate's Harris score exceeds the root**: replace the root with the candidate and sift down. The evicted root had the weakest score in the pool, so the pool now contains the K strongest features seen so far. Cost: $O(\log K)$.
3. **If the candidate's score is less than or equal to the root**: discard the candidate. Cost: $O(1)$.

Among candidates admitted by the 64-entry input FIFO, the heap retains at most the 1000 strongest Harris responses. The 1000-feature capacity is a design ceiling, not a per-frame pad. Global Top-K optimality under arbitrary unbounded bursts lies outside this bounded-admission model. The Top-K cadence test used 30,443 candidates, observed a maximum FIFO occupancy of 41, and recorded no drops for that trace.

5.3. Heap-Sort for Descending Order

After the frame's vertical synchronization (VSYNC) signal indicates that all candidates have been processed, the pool is sorted in-place using heap-sort to produce features in *descending* Harris order. Because the feature matcher processes features sequentially, this ordering prioritizes higher Harris-response features.

Parent–child relationships are implicit in the zero-based array: the parent of entry $i > 0$ is $\lfloor (i-1)/2 \rfloor$, and its children are $2i+1$ and $2i+2$. Sorting remains $O(K \log K)$, but synchronous Block-RAM access makes its cycle count larger than the simple comparison count. The Top-K cadence test measured 43,317 cycles (0.292 ms at 148.5 MHz) for the full 1000-entry sort. Isolated post-optimization synthesis mapped the Top-K block to 2878 CLB LUTs, 2304 registers, and 14 RAMB36 blocks, including ten RAMB36 blocks for the packed feature pool.

5.4. Integration with Matcher Preload

Once sorting completes, the sorted features and their 256-bit BRIEF descriptors are preloaded into the matcher (Section 6). A 64-deep FIFO decouples short bursts from the multi-cycle heap insertion FSM; a full FIFO drops an additional candidate rather than stalling the video stream. At the VSYNC boundary the matcher bank roles change, then Top-K sorts the completed pool. After the controller's fixed 3000-cycle wait, the stored features are presented to the matcher preload path and then to the builder preload path before the corresponding searches proceed.

5.5. Data Storage

Each packed pool entry contains an 11-bit row, 11-bit column, 256-bit BRIEF descriptor, and 71-bit Harris score, for a 349-bit record. The 1024-entry physical array is implemented

in Block RAM while the logical feature ceiling remains 1000. The 64-entry FIFO absorbs only short bursts. When it fills, later raster-order candidates are dropped before Top-K comparison, so the retained set can acquire spatial bias if dense bursts recur in particular image regions. No pre-Top-K spatial quota is implemented.

6. Feature Matching

6.1. Algorithmic Overview

Feature matching establishes correspondences between the two nearby frame slots presented to the matcher. For each feature in slot N, the matcher searches slot $N-1$ for the most similar BRIEF descriptor, as measured by Hamming distance (the number of differing bits between two 256-bit descriptors). A match is accepted only if the Hamming distance is at most the configured threshold of 106 out of 256 bits; this absolute threshold is a gate, not a guarantee that the accepted correspondence is geometrically correct.

The naive approach, comparing every feature in frame N against every feature in frame $N-1$, has $O(N^2)$ complexity. With $N = 1000$ features, this requires 1,000,000 descriptor comparisons per frame pair, each involving a 256-bit XOR and popcount. The spatial-bucket architecture exploits locality in video-rate imagery to reduce the comparison count to $O(N \cdot M)$ while the local-neighborhood occupancy M remains bounded. The symbol M is distinct from both the Top-K capacity and the camera intrinsic matrix $\mathbf{K}$.

6.2. Spatial Bucket Organization

The 1920×1080 image is partitioned into a grid of 32×32 pixel sub-buckets (using a 5-bit right-shift of pixel coordinates). The bucket grid is organized as 9 rows × 15 columns of 128×128-pixel cells, each subdivided into a 4×4 array of 32×32-pixel sub-buckets, for a total of $9 \times 15 \times 4 \times 4 = 2160$ sub-buckets (equivalently, a 36×60 grid of 32×32 sub-buckets; the bottom row is partially outside the 1080-pixel active area and remains empty). Each sub-bucket stores up to 15 features. A feature at pixel position (r, c) maps to sub-bucket $(\lfloor r/32 \rfloor, \lfloor c/32 \rfloor)$ with $O(1)$ address computation (a bit-shift operation in hardware).

The bucket directory is stored in a **Coordinate Directory RAM (CDRAM)** with 2160 logical entries, where each entry records the number of features in the bucket and a pointer to the first feature location. Its access pattern maps to LUTRAM. Feature data are stored in a **Crossbar Point RAM (CPRAM)** with 34,560 entries per bank (2160 buckets × 16 slots per bucket). Each 349-bit entry contains coordinates, descriptor, and score. The two CPRAM banks represent 24.12 Mbit of logical storage and map to Block RAM.

6.3. Ping-Pong Memory Banks

The matcher employs a **dual-bank ping-pong architecture**. Two complete copies of both CDRAM and CPRAM are maintained: bank A and bank B. The banks permit conflict-free separation between the matcher read role and builder write role. In the integrated schedule, however, the sorted-feature queries complete first and the same sorted features are then preloaded sequentially into the builder bank; these phases do not run concurrently. After initial baseline loading, a VSYNC-controlled advance can swap the bank roles.

The builder and matcher access different banks, preventing intentional same-bank read/write contention. The cost is duplicated on-chip storage: Block RAM for CPRAM and match lists and LUTRAM for the CDRAM directory.

6.4. Builder Pipeline

The builder inserts features into buckets through a 5-state FSM pipeline that respects BRAM's synchronous read latency:

1. **B_IDLE:** accept incoming feature, compute its bucket address via bit-shift, and issue the CDRAM read for that bucket's current entry count.
2. **B_ISSUE_CDRAM:** first cycle of the directory-memory read pipeline (address latched).
3. **B_WAIT_CDRAM:** second cycle of the directory-memory read pipeline (output-register delay).
4. **B_READ_CDRAM:** read the bucket entry count. If count < 15, advance to B_DO_APPEND. If count $= 15$, the feature is dropped (overflow tracked but gracefully handled) and the FSM returns to B_IDLE.
5. **B_DO_APPEND:** compute the CPRAM write address (bucket_index $\times$ 16 + count), write the feature's coordinates and descriptor to CPRAM, and write back the incremented bucket count to CDRAM.

6.5. Matching FSM

The matching controller declares four states, including `M_IDLE`; for each feature in the current frame, its three active processing phases are:

1. **M_READ_NEIGHBORS:** compute the feature's bucket position $(b_r, b_c) = (\lfloor r/32 \rfloor, \lfloor c/32 \rfloor)$. Enumerate the 9 surrounding buckets in a 3×3 neighborhood (from $(b_r - 1, b_c - 1)$ to $(b_r + 1, b_c + 1)$). For each bucket, read its CDRAM entry to determine how many candidate features it contains.
2. **M_STREAM_CANDIDATES:** for each candidate feature in the nine neighboring buckets, read its data from CPRAM and compute the Hamming distance between the query's 256-bit descriptor and the candidate's descriptor. The fixed 3×3 neighborhood supplies the implemented spatial bound; the configured radius value does not further reduce this candidate set.
3. **M_FLUSH_PIPELINE:** after all candidates are evaluated, register the best match when its minimum Hamming distance is at most 106.

6.6. Pipelined Hamming Distance Computation

The Hamming distance between two 256-bit descriptors is computed by XOR followed by population count (popcount). In the functional RTL schedule, CPRAM presents one candidate per cycle. The 256-bit XOR is divided into four 64-bit segments and accumulated through stages P5a–P5d; stages P6–P8 apply the threshold and update the best candidate. After pipeline fill, the implemented single-stream datapath accepts **one complete candidate-distance evaluation per cycle** and requires one CPRAM read per cycle.

6.7. Match Classification

The RTL also records whether an accepted match exceeds 60 pixels in either image axis. This is an image-motion indicator, not the steady-state bank-advance or solve-enable condition: after initialization, bank roles advance at VSYNC boundaries. The indicator cannot distinguish translational parallax from rotation, depth-dependent optical flow, a moving object, or an outlier and is not treated as a triangulation-validity test.

The identical-image repeatability test produces 999 self-matches with near-zero Hamming distances, confirming stable detector, descriptor, and matcher behavior for that stimulus.

6.8. Complexity Analysis

For $N = 1000$ features per frame and an average of M features per 3×3 bucket neighborhood:

Table 6 quantifies the reduction in descriptor comparisons at the 1000-feature design ceiling.

Table 6. Descriptor comparisons per frame, brute-force vs spatial-bucket matcher.

Approach	Comparisons per Frame	At $N = 1000$, $M = 75$
Brute-force	$N^2 = 1{,}000{,}000$	1,000,000
Spatial-bucket	$N \cdot M \approx 1000 \times 75$	75,000

At the illustrative $M = 75$ point, the formula gives 75,000 rather than 1,000,000 comparisons, a projected 13.3× count reduction. The value of M is scene-dependent; the asymptotic growth is linear in the query-feature count only while local bucket occupancy remains bounded.

6.9. Data Storage

Table 7 summarizes the matcher's two-bank storage cost.

Table 7. Storage used by the feature matcher.

Structure	Medium	Size	Purpose
CDRAM (×2 banks)	Distributed RAM (LUTRAM)	2160 entries × 2 = 4320 entries	Bucket directory; requested BRAM style falls back to LUTRAM for the implemented access pattern.
CPRAM (×2 banks)	BRAM	69,120 entries × 349 bits = 24.12 Mbit	Feature coordinates, 256-bit descriptors, and scores.
Match list (×2 banks)	BRAM	1000 entries × 2 = 2000 entries	Matched coord. pairs (prev./curr. row/col) + Hamming.
Candidate pipeline registers	FFs	One 256-bit candidate stream	Four-stage 64-bit-segment popcount; one completed Hamming distance per clock after fill.

Figure 3 illustrates the 60-column × 36-row grid and the 3×3 neighborhood examined for each query.

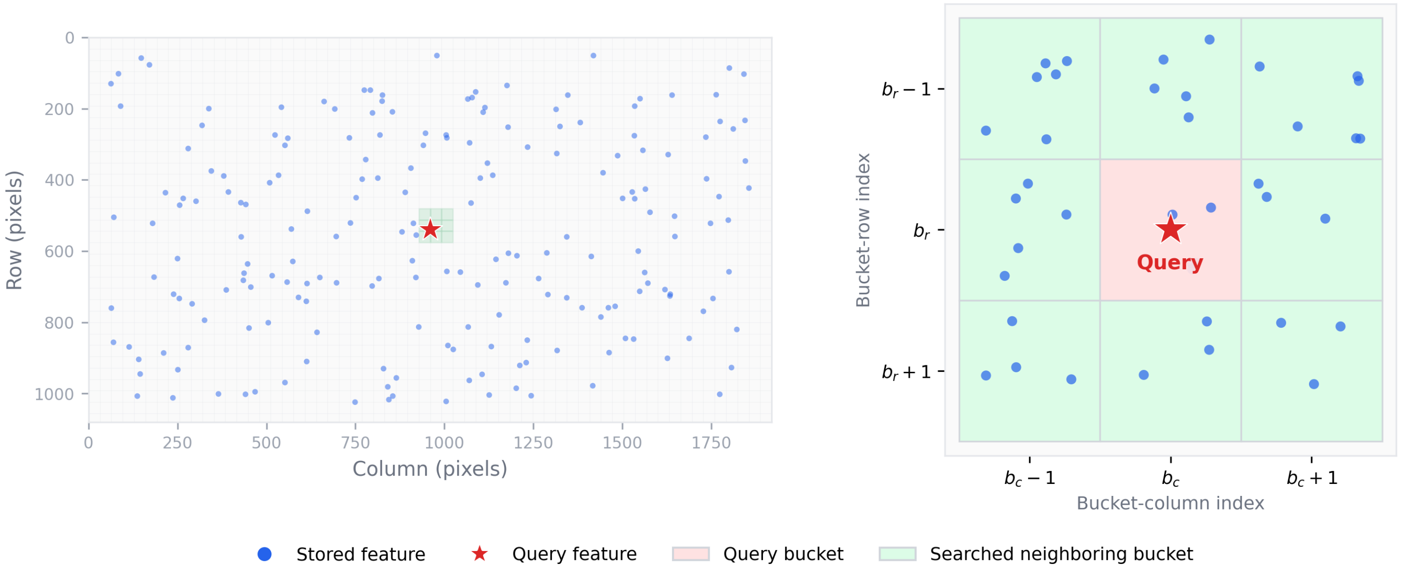

Figure 3. Spatial-bucket matching architecture. Left axes are image column and row in pixels (px); blue dots are stored candidate features and the red star identifies one query location within the highlighted local grid. A 1920×1080 image is partitioned into 60 columns ×36 rows of 32×32-pixel sub-buckets. Right axes are dimensionless bucket-column and bucket-row indices: b_c and b_r denote the query bucket indices, the red star the query, the pale-red cell its bucket, and the pale-green cells the fixed 3×3 search neighborhood. The neighborhood limits candidates before 256-bit Binary Robust Independent Elementary Features (BRIEF) Hamming-distance comparison; no additional radius filter reduces the shown set.

7. Geometric Pipeline

The `system_controller` orchestrates the six-stage geometry back end, which maps matched 2D feature correspondences to point-coordinate output records. Its `sfm_controller` sub-module manages the two-pass solve–prune–resolve strategy and coordinates data flow among the geometry sub-modules.

7.1. Coordinate Normalization

7.1.1. Algorithm

Pixel coordinates must be converted to K-normalized coordinates before geometric computation. The camera intrinsic matrix is

$$K = \begin{bmatrix} f_x & 0 & c_x \\ 0 & f_y & c_y \\ 0 & 0 & 1 \end{bmatrix}, \tag{1}$$

and a pixel at (u, v) is normalized according to

$$\hat{x} = \frac{u - c_x}{f_x}, \qquad \hat{y} = \frac{v - c_y}{f_y}. \tag{2}$$

Equations (1) and (2) apply K^{-1} and convert pixels to calibrated, dimensionless normalized camera coordinates. This operation is distinct from Hartley's numerical preconditioning [18]: inside the HLS solver, each calibrated point set is subsequently centered and isotropically scaled so that its RMS distance from the centroid is $\sqrt{2}$.

7.1.2. Hardware Implementation

The coordinate normalizer is a 3-stage pipeline:

- **Stage 1 (P1):** convert pixel coordinates from unsigned integers to sfixed format. Extract principal point (c_x, c_y) from the K matrix.
- **Stage 2 (P2):** subtract the principal point: $u' = u - c_x$, $v' = v - c_y$.
- **Stage 3 (P3):** multiply by precomputed reciprocal focal lengths: $\hat{x} = u' \times (1/f_x)$, $\hat{y} = v' \times (1/f_y)$. The reciprocals use the wider `sfixed(3,-28)` format (28 fractional bits), and the result is resized to `sfixed(19,-12)` for downstream consumption.

Two instances of the normalizer operate in parallel within `sfm_controller`, one for each point in a match pair, providing normalized coordinates for both the previous-frame and current-frame features simultaneously. Latency: 3 clock cycles per coordinate pair.

7.1.3. Camera Parameters

The system uses calibrated intrinsic parameters: $f_x = 1735.037$, $f_y = 1736.430$, $c_x = 956.295$, $c_y = 541.765$ (for 1920×1080 resolution). These constants are hardcoded into the VHDL design following an offline calibration procedure using Zhang's method [32].

7.1.4. Data Storage

Pipeline registers (P1 through P3 validity flags and intermediate values) are stored in **flip-flops**. The reciprocal focal length constants ($1/f_x$ and $1/f_y$) are `sfixed(3,-28)` constants synthesized as combinational logic. DSP slices are used for the multiplication in stage P3.

7.2. Eight-Point Algorithm (Two-Pass Architecture)

7.2.1. Algorithm

The eight-point algorithm [19], with Hartley's normalization [18], recovers the essential matrix $\mathbf{E}$ directly from point correspondences. Given n matched pairs $(\hat{\mathbf{x}}_i, \hat{\mathbf{x}}'_i)$ in K-normalized camera coordinates, where the unprimed point is from the previous frame $(N-1)$ and the primed point is from the current frame (N), each pair contributes the following row to the coefficient matrix $\mathbf{A}$:

$$\mathbf{A}_i = [x'_i x_i,\ x'_i y_i,\ x'_i,\ y'_i x_i,\ y'_i y_i,\ y'_i,\ x_i,\ y_i,\ 1]. \quad (3)$$

Equation (3) supplies the rows whose smallest right-singular vector is reshaped into the 3×3 essential-matrix estimate. As a hardware optimization, the estimate is projected to rank 2 by zeroing the smallest singular value. The downstream pose recovery extracts $\mathbf{R}$ and $\mathbf{t}$ from the singular vectors $\mathbf{U}$ and $\mathbf{V}^T$ [1]; the controlled numerical audits in Section 10.5 characterize the resulting fixed-point operating envelope.

7.2.2. Two-Pass Strategy

The `sfm_controller` implements the two-pass strategy with an 18-state FSM. Its states sequence registered match-list reads, coordinate normalization, A-matrix formatting, the first solve, pruning, conditional inlier reloading, the second solve, and completion.

Pass 1 (all matches):

1. **Read matches:** load match coordinates from the `sfm_controller`'s local BRAM copy. That copy originates in the matcher's completed bank and is transferred through the processing-module and system-controller match-list banks before geometry starts. The local read has a 3-cycle latency (two wait states after address issue), accommodated by the `READ_MATCH_WAIT` / `READ_MATCH_WAIT2` states.
2. **Normalize:** feed both match coordinates through dual coordinate normalizer instances (3-cycle pipeline each).

3. **Format:** stream normalized coordinates to the `eight_point_formatter`, which constructs A matrix rows from the Kronecker-product-style encoding.
4. **Accumulate:** store the complete A matrix (up to 1000 rows $\times$ 9 columns $\times$ 32 bits).
5. **Solve:** trigger the HLS eight-point solver kernel, which reads the A matrix from the AXI memory endpoint, forms $\mathbf{G} = \mathbf{A}^T\mathbf{A}$, extracts the minimum-eigenvalue direction by inverse power iteration, applies rank-2 projection, and returns the 3×3 matrix estimate.

Pruning (Section 7.3): identifies inlier matches using algebraic epipolar error.
Pass 2 (inliers only):

6. **Re-read:** iterate through matches again, but skip outliers using the inlier bitmask from pruning.
7. **Re-normalize and re-format:** process only inlier matches.
8. **Re-solve:** when at least eight inliers remain, call the HLS solver again with the reduced system.

The second pass uses all retained inliers rather than selecting a separate minimal subset. With exactly eight inliers the system is determined; with more than eight it is over-determined. Its objective is the algebraic residual before rank-2 projection, distinct from geometric reprojection or Sampson error.

7.2.3. Data Storage

The A matrix is the largest data structure in the geometry pipeline: 1000 rows $\times$ 9 columns $\times$ 32 bits = 288,000 bits. It is stored in **nine BRAM banks** (one per A-matrix column, declared with `ram_style = "block"` in `sfm_controller.vhd`), so that an entire row of nine elements is read in a single cycle by addressing all nine banks in parallel. Match coordinates are also BRAM-resident. A completed matcher bank is copied into a processing-module match-list bank, then into a system-controller bank, and finally into the `sfm_controller`'s local previous/current row/column banks; this staged handoff decouples list ownership but adds copy latency. The wrapper streams the A matrix row-by-row over AXI4 to the solver-visible memory endpoint and reads the returned 3×3 matrix from that endpoint. In the implemented VCU118 design, this transaction space is a 128 KiB on-chip BRAM surrogate rather than external DDR (Section 8).

7.3. Algebraic Pruning

7.3.1. Algorithm

For each match pair $(\hat{\mathbf{x}}, \hat{\mathbf{x}}')$ and the estimated essential matrix $\mathbf{E}$, the algebraic epipolar error is

$$e = |\hat{\mathbf{x}}'^T \mathbf{E} \hat{\mathbf{x}}|. \quad (4)$$

A match is classified as an inlier when the error in Equation (4) satisfies $e < \tau$, where τ is scaled by the magnitude of $\mathbf{E}$: $\tau = 0.10 \times \max_{i,j} |E_{i,j}|$. This normalization makes the decision invariant to the arbitrary scale of the homogeneous matrix estimate; it is not itself a measure of matrix conditioning.

7.3.2. Why Algebraic Pruning Instead of RANSAC

RANSAC [20] is the standard outlier rejection method in software SfM. It can be implemented with bounded latency by fixing the iteration cap and sampling sequence. In the present architecture, however, repeatedly invoking the existing solver would exceed the frame budget and require substantially more control and memory-access logic:

1. **Random sampling** requires a pseudo-random number generator and random-access indexing into the match list, complex control logic with unpredictable memory access patterns.
2. **Iterative re-fitting** requires hundreds of repeated minimal-set model fits and full-list inlier evaluations. This workload is substantially larger and less regular than the two deterministic over-determined solves used here.
3. **Iteration control** normally uses confidence-driven early termination, which introduces variable latency; a fixed cap restores a deterministic bound at the cost of always provisioning for that worst case.

The two-pass method is intended only for selected nearby pairs in which sufficient overlap, predominantly static content, and descriptor/spatial gating already produce a high initial inlier ratio. Under that assumption the first over-determined estimate can be useful enough to support one pruning pass. When the assumption is violated, the first estimate can be corrupted and the pruner has no independent robust hypothesis from which to recover.

7.3.3. Hardware Implementation

The pruner is a 6-stage pipelined datapath with a 4-state control FSM:

- **P1:** K-normalize pixel coordinates (using the same reciprocal focal length constants as the coordinate normalizer).
- **P2:** compute initial products $E_{0j} \cdot \hat{x}^{(N-1)}$, $E_{0j} \cdot \hat{y}^{(N-1)}$ for row 0 of **E**.
- **P3:** accumulate row 0 sum: $r_0 = E_{00}\hat{x}^{(N-1)} + E_{01}\hat{y}^{(N-1)} + E_{02}$. Similarly for rows 1 and 2.
- **P4:** multiply by second point: $\hat{x}^{(N)} \cdot r_0$, $\hat{y}^{(N)} \cdot r_1$, r_2.
- **P5:** final summation: $e = |\hat{x}^{(N)}r_0 + \hat{y}^{(N)}r_1 + r_2|$.
- **P6:** registered absolute value and threshold comparison. Output: inlier bit and updated count.

After pipeline priming, the pruner produces one inlier decision per clock cycle: the FSM's `S_CALC` state feeds one new match every clock with no stall or back-pressure condition, and the 6-deep `p1_valid...p6_valid` chain drains in roughly six further cycles. For 1000 matches, the datapath requires approximately 1006 feed/drain cycles; including FSM boundaries gives approximately 1008 clocks from start assertion to completion, or 0.0068 ms at 148.5 MHz.

7.3.4. Data Storage

The E matrix is latched in **FFs** at the start of pruning. The inlier bitmask (1000 bits) and inlier count are stored in FFs. All pipeline stage registers (P1–P6 valid flags and intermediate computation values) are FFs.

7.4. Essential Matrix Extraction

Because the eight-point solver receives dimensionless K-normalized camera coordinates, its output natively satisfies the calibrated epipolar constraint $\hat{\mathbf{x}}'^T\mathbf{E}\hat{\mathbf{x}} = 0$. Thus, the solver directly estimates the essential matrix **E**; no additional computation $\mathbf{E} = K^T\mathbf{F}K$ is needed. This essential matrix is then propagated to the downstream pose recovery stage. Some internal VHDL signals retain the name `F_matrix`, but they carry this calibrated essential-matrix estimate.

7.5. Pose Recovery via SVD

7.5.1. Algorithm

The essential matrix $\mathbf{E}$ encodes the relative rotation $\mathbf{R}$ and translation direction $\mathbf{t}$ between two camera views. Recovery proceeds by the SVD [1]

$$\mathbf{E} = \mathbf{U}\mathbf{\Sigma}\mathbf{V}^T. \tag{5}$$

Using Equation (5), four candidate poses are then constructed:

$$\mathbf{R}_1 = \mathbf{U}\mathbf{W}\mathbf{V}^T, \quad \mathbf{R}_2 = \mathbf{U}\mathbf{W}^T\mathbf{V}^T, \quad \mathbf{t} = \pm\mathbf{U}_{:,3}, \tag{6}$$

where $\mathbf{W} = \begin{bmatrix} 0 & -1 & 0 \\ 1 & 0 & 0 \\ 0 & 0 & 1 \end{bmatrix}$. Equation (6) leaves a four-way sign/rotation ambiguity that is resolved by the cheirality criterion.

In non-degenerate two-view geometry, the physically valid pose is the candidate that places triangulated scene points in front of both cameras (the **cheirality criterion**). The implemented pose-selection RTL evaluates a division-free depth-sign test using one registered correspondence, specifically match-list entry 0, and selects the first valid candidate in a fixed priority order.

7.5.2. Hardware Implementation

Pose recovery uses five active control phases. `S_IDLE` starts the SVD directly, `S_WAIT_SVD` handles its response, `S_STAGE1_SETTLE` provides the implemented five-cycle wait, `S_CALC_AND_CHECK` builds and evaluates the candidates, and `S_DONE` completes the transaction. The fixed candidate/cheirality datapath is:

1. **SVD call:** the 3×3 essential matrix is decomposed by the pure-VHDL `svd_3x3` module, which performs CORDIC-based Jacobi eigendecomposition with signed 32-bit Q8.24 internal arithmetic, producing U, S, and V^T through direct register I/O. Negative vectoring inputs are folded into the CORDIC right half-plane. The recovered singular values are then fully ordered in descending order while the same swaps are applied to the corresponding columns of $\mathbf{V}$. The smallest singular direction therefore occupies array index 2. When the norm of $\mathbf{U}_{:,3}$ is below the fixed degeneracy threshold, that column is completed as $\mathbf{U}_{:,1} \times \mathbf{U}_{:,2}$.
2. **Stage 1:** if $\det(\mathbf{U})\det(\mathbf{V}^T) < 0$, negate the fully ordered null-direction column at index 2 of $\mathbf{U}$ before forming $\mathbf{A}_1 = \mathbf{U}\mathbf{W}$ and $\mathbf{A}_2 = \mathbf{U}\mathbf{W}^T$.
3. **Stage 2:** compute $\mathbf{R}_1 = \mathbf{A}_1\mathbf{V}^T$, $\mathbf{R}_2 = \mathbf{A}_2\mathbf{V}^T$, and extract $\mathbf{t}_0 = \mathbf{U}_{:,3}$. Both signs of $\mathbf{t}_0$ are evaluated in the cheirality stage, so the sign induced by the handedness correction is immaterial.
4. **Cheirality datapath:** K-normalize the single registered sample correspondence and project it through all four candidates. The signs of its two depths are evaluated without explicit triangulation division.
5. **Selection:** choose the first candidate that places the sample in front of both cameras, in the deterministic order $(\mathbf{R}_1, +\mathbf{t})$, $(\mathbf{R}_1, -\mathbf{t})$, $(\mathbf{R}_2, +\mathbf{t})$, $(\mathbf{R}_2, -\mathbf{t})$; if none passes, the RTL falls back to the first candidate. Output: $\mathbf{R}$ (3×3) and $\mathbf{t}$ (3-vector).

The single-sample policy is inexpensive and deterministic but is sensitive to a noisy, mismatched, or geometrically weak first correspondence. A retained-list vote, robust support test, or explicit triangulation-angle gate is therefore identified as a future refinement.

7.5.3. Data Storage

SVD output matrices (U, V^T, each 3×3), candidate matrices, and the fixed cheirality datapath's intermediate values are stored in **FFs**. The K matrix and one sample correspondence are latched at the start of the computation; the system controller supplies match-list entry 0 through a registered BRAM read.

7.6. DLT-Derived Inhomogeneous Linear Triangulation

7.6.1. Algorithm

Given the recovered pose $(\mathbf{R}, \mathbf{t})$ and a matched pair of K-normalized points $(\hat{\mathbf{x}}_1, \hat{\mathbf{x}}_2)$, the 3D point is recovered using a fixed-point inhomogeneous solver derived from the standard DLT equations [1]. The two projection matrices are

$$\mathbf{P}_1 = [\mathbf{I} \mid \mathbf{0}], \qquad \mathbf{P}_2 = [\mathbf{R} \mid \mathbf{t}]. \tag{7}$$

For each match, the projection matrices in Equation (7) define the standard homogeneous system $\mathbf{A}\mathbf{X}_h = \mathbf{0}$. The hardware selects the finite-point affine chart $\mathbf{X}_h = (X, Y, Z, 1)^T$, partitions $\mathbf{A} = [\mathbf{C} \mid \mathbf{a}_4]$, and solves

$$\min_{\mathbf{X}} \|\mathbf{C}\mathbf{X} + \mathbf{a}_4\|_2^2, \qquad \mathbf{C}^T\mathbf{C}\mathbf{X} = -\mathbf{C}^T\mathbf{a}_4. \tag{8}$$

Gaussian elimination with partial pivoting is applied to this 3×3 normal-equation system. This is an inhomogeneous linear least-squares triangulator rather than a null-space homogeneous DLT/SVD solve. Forming $\mathbf{C}^T\mathbf{C}$ approximately squares the condition number of $\mathbf{C}$ and can amplify fixed-point error. The method assumes a finite solution in the selected chart; the controlled audit therefore includes low-angle and poorly conditioned cases and reports the measured numerical boundary. The subsequent "normalization" state refers strictly to fixed-point radix realignment after signed 32-bit Q8.24 arithmetic.

7.6.2. Hardware Implementation

The triangulation stage consists of two modules:

- `triangulator`: a seven-phase loop controller that starts the solver directly after coordinate normalization. The controller iterates over up to 1000 retained matches, constructs $\mathbf{P}_1$ and $\mathbf{P}_2$, invokes the linear solver, and emits address/write-enable/X/Y/Z events.
- `trig_dlt_solver`: the source module implementing Equation (8) with a 7-state FSM. It constructs the 4×4 $\mathbf{A}^T\mathbf{A}$ matrix, applies Gaussian elimination with partial pivoting to its top-left 3×3 block and fourth-column right-hand side, back-substitutes with the homogeneous coordinate fixed to one, and realigns the result. Nine arithmetic-heavy steps each hold for $W = 12$ clocks. Schedule validation reports 111 solver clocks; retaining nine controller clocks gives a conservative 120-clock per-point term in the latency model.

The linear solver uses the same two-scale integer arithmetic as the SVD module (Section 8.3): signed 32-bit Q20.12 interfaces and signed 32-bit Q8.24 internally for Gaussian elimination and back-substitution. The additional fractional bits preserve smaller coordinate differences than the interface format, while the fixed 32-bit word length establishes the range and conditioning envelope evaluated in Section 10.5.

The divider's 64-bit intermediate quotient is limited explicitly to raw words `0x3FFFFFFF` and `0xC0000000`, corresponding to $+64 - 2^{-24}$ and -64 in Q8.24. Other arithmetic realigns to a 32-bit result. If a divisor passed to the divider is exactly zero, the divider returns zero; the elimination and back-substitution states additionally guard their pivot tests, skip the affected elimination update, or assign the corresponding solution component zero. The

current interface encodes this guarded zero-pivot fallback as zero; deployments requiring explicit point-validity signalling can add a validity flag at the receiver interface.

7.6.3. Data Storage

The triangulator exposes an 11-bit address, write enable, and three 32-bit coordinates; storage belongs to the receiver. The 1000-point ceiling corresponds to a maximum coordinate payload of 12,000 bytes, but the core does not instantiate a BRAM-backed point-cloud array. Although a generic signed 32-bit Q8.24 word spans $[-128, +128)$, this implementation's divider limits its own quotient outputs to $[-64, +64 - 2^{-24}]$. We therefore call occurrences of those values *divider-clamp hits*, not generic Q8.24 representation endpoints. Internal matrices and intermediate values are held in registers.

8. Accelerator Integration

8.1. Rationale for Mixed Implementation

The eight-point solver uses float32 arithmetic to compute the null direction of a 1000×9 system through inverse power iteration with Cholesky factorization. It is implemented in C++ and synthesized with Vitis HLS into an RTL module with AXI4 master interfaces for memory access and AXI-Lite slave interfaces for control-register configuration. This is the pipeline's only HLS-generated arithmetic block; the surrounding stages are implemented in VHDL. The mixed implementation concentrates floating-point arithmetic in the large matrix solve while retaining cycle-explicit fixed-point RTL for the remaining geometry stages.

The 3×3 SVD decomposition for pose recovery uses a pure-VHDL CORDIC-based Jacobi eigensolver (Section 8.3), avoiding a second floating-point kernel. The module-level XSim audits in Section 10.5 pass all 72 scored nondegenerate temporal-envelope cases and all 36 planar/homography-dominant stress cases. The deliberately ill-conditioned stress cases define the boundary of the tested numerical envelope.

8.2. Eight-Point Solver Kernel

The HLS kernel (`eight_point_solver_kernel`) implements:

1. **Hartley normalization:** the input coordinates are further normalized by isotropic scaling such that the RMS distance from the centroid equals $\sqrt{2}$ [18]. Transformation matrices $\mathbf{T}_1$ and $\mathbf{T}_2$ are stored for denormalization.
2. **Gram matrix:** $\mathbf{G} = \mathbf{A}^T\mathbf{A}$ (9×9 symmetric).
3. **Inverse power iteration:** 24 iterations with Cholesky factorization ($\mathbf{LL}^T$ decomposition) to find the eigenvector corresponding to the smallest eigenvalue. Ridge regularization ($\epsilon = 10^{-2}$) ensures positive definiteness.
4. **Rank-2 projection:** the 3×3 matrix is decomposed via Jacobi eigendecomposition (12 sweeps), and the smallest singular value is zeroed.
5. **Denormalization:** $\mathbf{E} = \mathbf{T}_2^T \mathbf{E}_n \mathbf{T}_1$.

The kernel operates entirely in `float` (32-bit IEEE 754), reads up to 9000 floats (1000×9 matrix, matching the Top-K cap and the wrapper's `A_ROWS` generic), and writes 9 floats (the 3×3 E matrix). In the implemented design it runs at **49.5 MHz**, derived from the 148.5 MHz pixel clock by an on-chip MMCM divide-by-3; AXI clock converters bridge control and data between the domains. Final post-route timing analysis covers both physical clocks and meets timing. A same-clock simulation records approximately 258,000 wrapper polling events between `ap_start` and observation of `ap_done`. Evaluating that count at 49.5 MHz gives the **nominal 5.21 ms per-invocation term** used in the analytical timing model.

The implemented memory map places the A-matrix input at byte address `0x00000000` and the returned matrix at `0x00001000`. A 1000×9 float32 A matrix occupies 36,000 bytes,

so these address ranges overlap. The HLS C++ kernel copies all requested A words into a local array before writing its nine-word result; input and output therefore occupy non-overlapping transaction phases.

8.3. SVD 3×3 Decomposer: Pure-VHDL CORDIC Jacobi Implementation

The 3×3 SVD decomposition for pose recovery is implemented entirely in pure VHDL using a CORDIC-based Jacobi eigensolver. This module (`svd_3x3`) has Q20.12 matrix interfaces and uses signed 32-bit Q8.24 internally; "24" denotes the fractional-bit count, not a 24-bit word. It requires no floating-point hardware, memory transaction, or AXI bus access. The implementation proceeds as follows:

1. **Frobenius normalization:** scale **E** by $1/\|\mathbf{E}\|_F$ using a Newton–Raphson reciprocal-square-root approximation to keep eigenvalues $O(1)$, critical for convergence of the Jacobi iterations with fixed-point tolerance.
2. **Compute $\mathbf{E}_n^T\mathbf{E}_n$** (symmetric 3×3) using signed 32-bit Q8.24 arithmetic.
3. **CORDIC-based Jacobi eigendecomposition:** iterative Jacobi sweeps using CORDIC rotations to diagonalize the symmetric matrix, yielding the right singular vectors **V** and squared singular values λ_i. Each sweep applies Givens rotations to the three off-diagonal pairs; a negative vectoring abscissa is folded into the CORDIC right half-plane before the half-angle rotation is formed.
4. **Singular values:** clamp negative fixed-point round-off at zero and recover the original scale as $\sigma_i = \|\mathbf{E}\|_F\sqrt{\max(\lambda_i, 0)}$. The square root is evaluated as $\lambda_i\lambda_i^{-1/2}$ using a Newton–Raphson reciprocal-square-root approximation.
5. **Left singular vectors (U) recovery:** form each unnormalized column $\tilde{\mathbf{U}}_i = \mathbf{E}_n\mathbf{V}_i$, compute its squared norm, and normalize it using seven effective Newton–Raphson reciprocal-square-root updates after the initial estimate. For nonzero singular values this is equivalent to division by $\sqrt{\lambda_i}$, while avoiding a general divider and avoiding any ambiguity about diagonal regularization.
6. **Complete singular-value ordering:** compare the three recovered singular values, move the maximum into array index 0, order the remaining values at indices 1 and 2, and apply every swap to the corresponding columns of **V**. The smallest recovered singular direction is therefore placed at index 2.
7. **Conditional null-basis completion:** after computing and normalizing all three columns of $\mathbf{U} = \mathbf{E}_n\mathbf{V}$, test the fully ordered column at index 2. If its squared norm is below the fixed threshold, replace that column with the cross product of columns 0 and 1; otherwise retain the normalized column.

The module accepts 9 elements of the E matrix through direct register I/O and produces 21 outputs (U: 9, S: 3, V^T: 9). Across the module-level audits, the observed start-to-output schedule ranges from 175 to 451 clocks; the latency model retains 531 clocks as a conservative combined SVD-plus-pose term. All 72 scored nondegenerate temporal-envelope cases pass their predeclared thresholds, including all 24 forward-motion cases. A separate stress audit passes all 36 planar/homography-dominant cases, while deliberately ill-conditioned matrices define the boundary of the tested envelope.

8.4. VHDL Wrapper Design Pattern

The HLS eight-point solver is encapsulated by a VHDL wrapper that sequences the required AXI transactions. The implemented pattern is:

1. **sfixed → float32 conversion:** on `start_in`, the wrapper latches only the row count and memory-base addresses. It then accepts one 9-element A-matrix row at a time, converts that row from `sfixed(19,-12)` to IEEE 754 `float(8,-23)` with VHDL-2008's

`to_float()` function, and holds the nine converted words in a row buffer for the following burst. There is no bulk matrix conversion or full-matrix register latch.

2. **AXI burst write:** the converted float32 values are written to the memory endpoint in one 9-beat INCR burst per A-matrix row (`AWLEN`=8), for up to 9000 float words.
3. **AXI-Lite kernel configuration:** the wrapper writes the memory input and output pointer addresses and any scalar parameters to the HLS kernel's control registers via AXI-Lite writes.
4. **Kernel launch and polling:** the wrapper asserts `ap_start` by writing to the `AP_CTRL` register, then polls the `ap_done` bit. A 500,000-count guard bounds AXI-Lite polling and returns through the ordinary completion interface.
5. **AXI burst read:** the resulting matrix is read back from the memory endpoint.
6. **float32 $\rightarrow$ sfixed conversion:** the read float32 values are converted back to Q20.12 using `to_sfixed()` and presented on the wrapper's output ports.

The wrapper FSM is sequential. AXI `BRESP`/`RRESP` values and the polling guard return through the ordinary completion interface. The SVD and triangulator use direct-register interfaces and require no such wrapper.

9. Inter-Frame Pipeline Scheduling

9.1. Timing Architecture

The intended inter-frame schedule overlaps pixel processing with geometry computation. The streaming pixel front-end and the sequential geometry back-end can execute concurrently on different frame slots; Section 9.3 details the stages that continue after the current correspondence list is complete.

9.2. Front-End Work Associated with One Input Frame

At the intended 1920×1080, 60 fps input format, one frame period is 16.67 ms at a pixel clock of 148.5 MHz. The RTL associates the following streaming and post-VSYNC work with each input frame:

1. **Pixel streaming:** FAST-12 detection, Harris scoring, and 256-bit BRIEF descriptor computation, fully pipelined at one accepted active pixel per clock. The active image contains 2,073,600 pixels; the complete 1080p60 raster contains 2,475,000 link-clock cycles including blanking.
2. **Top-K selection:** $O(\log K)$ per candidate, fully overlapped with pixel streaming.
3. **Post-VSYNC front-end handoff:** the completed Top-K pool is sorted and the controller observes its fixed 3000-cycle (20.2 μs) post-sort interval. This interval covers clearing the matcher's 2160-entry write-bank CDRAM and retains additional margin.
4. **Matching and baseline preload:** the newly sorted features are first presented to the matcher against its current read bank and are then written sequentially into the builder bank for a subsequent interval.

9.3. What Continues after Match-List Handoff

The front-end matching workflow and the geometry-launch workflow are distinct. The former matches a newly sorted Top-K pool and then preloads the builder bank. The latter waits for a completed match-list bank: after a bank-advance pulse is latched, the next VSYNC swaps the match-list read role; the master controller then latches that completed bank's count, copies its entries through the 3-cycle BRAM read path, and starts geometry. Geometry can overlap pixel processing of a later frame, but it does not consume the list currently being written. Counts below are reported at the **1000-match design ceiling**. For reference, one testbench pair (frame_0000 vs frame_0017) produces 940 matches, a scene-dependent observation rather than a hardware limit. The reported times form an

analytical recombination: VHDL-side counts are evaluated at 148.5 MHz and solver counts at the intended 49.5 MHz kernel period.

- **Eight-point solver kernel (Pass 1):** a same-clock simulation records approximately 258,000 wrapper polling events. Treating one event as one 49.5 MHz kernel period gives the analytical **5.21 ms** term.
- **Algebraic pruning:** one match is fed per clock into a 6-stage pipeline. The approximately 1006 feed/drain cycles, or approximately 1008 start-to-done clocks, contribute **0.0068 ms**.
- **Eight-point solver kernel (Pass 2):** the same nominal assumption gives **5.21 ms**.
- **SVD and pose recovery:** the fixed-point SVD tests observe 175–451 clocks from SVD start to output; a schedule replay observes 20 pose-controller clocks when an SVD response is supplied immediately. The latency model retains a conservative combined term of 531 `pix_clk` cycles = **0.004 ms**.
- **Triangulation:** up to 1000 points at the design ceiling, using 111 solver clocks plus a conservative nine controller clocks per point: **120,000 `pix_clk` cycles = 0.81 ms**.
- **Nominal match loading, wrapper control, memory, and AXI allowance** (flat-buffer load, A-matrix streaming, start/done handshakes, and result read-back): the analytical model assigns ∼280,000 `pix_clk` cycles, or **1.89 ms**.

The exact generated AXI4 and AXI-Lite clock converters were exercised directly at 148.5 MHz and 49.5 MHz across six relative clock phases: 768 completed transactions produced 3072 recorded latency samples. The largest observed nine-beat AXI4 service time was 427.609 ns. The FIFO model omits synchronizer delay; adding three destination and three source clock cycles gives a conservative 508.417 ns per transaction. The wrapper serializes one nine-beat write per solver row and one nine-beat read per pass, so two passes perform $M + P + 2 \leq 2002$ converter transactions at $P \leq M \leq 1000$. At that ceiling, the observed-to-conservative converter-service envelope is 0.856–1.018 ms. This always-ready isolated test excludes SmartConnect and Block-RAM arbitration, memory stalls, kernel service, and board traffic.

Nominal geometry-back-end subtotal at the 1000-match design ceiling: under the assumptions above, the arithmetic sum is approximately **13.13 ms**, dominated by two nominal HLS solver terms (10.42 ms combined), with the wrapper/memory/AXI allowance (1.89 ms), triangulation (0.81 ms), algebraic pruning, and the conservative SVD-plus-pose term. The measured converter envelope is reported separately because the 1.89 ms allowance already includes AXI service. The 13.13 ms value is an analytical geometry-back-end subtotal, not an on-board latency measurement. It excludes the active-video raster, Top-K sorting, the fixed 3000-cycle post-sort interval, matcher/preload handoff, board I/O, and point-record reception.

Feature matching and Top-K sorting are outside the 13.13 ms subtotal. The measured Top-K trace takes 43,317 clocks (0.292 ms) to sort. Representative matcher service time belongs to the end-to-end deployment characterization.

9.4. Pipeline Overlap

The intended steady-state schedule allows two stored intervals to be in flight across the system's major domains:

- **Current raster:** pixels stream through detection while features accumulate in Top-K.
- **Earlier completed frame-pair bank:** the geometry pipeline executes solver, pruning, re-solve, SVD, pose selection, and triangulation to emit signed point-coordinate records.

This dataflow permits the streaming front end and sequential geometry back end to work on different stored intervals. Matching and builder preload complete a new list, while

the master controller later copies a completed read-bank list and launches geometry after a subsequent VSYNC.

9.5. The VSYNC Boundary

The front-end and master controllers use VSYNC for related but separate actions:

1. The front-end controller freezes and heap-sorts the completed Top-K pool, then observes the fixed 3000-cycle post-sort interval while the matcher's 2160-entry write-bank CDRAM is cleared, with additional margin.
2. Sorted features are matched against the matcher's current read bank.
3. The same sorted features and descriptors are subsequently preloaded into the builder bank.
4. A displacement request can be recorded when an accepted match exceeds 60 pixels on either axis, but after initial baseline loading it is not the sole condition for the matcher's VSYNC bank advance.
5. Separately, when the master controller has latched a bank-advance pulse, the following VSYNC exposes the completed match-list bank for reading.
6. After one settling clock, the master controller latches the completed match count, copies each entry through the 3-cycle read path, and then starts geometry on that copied list.

9.6. Scope of the Composite Timing Projection

The approximately 13.13 ms analytical subtotal identifies the two HLS-solver terms as dominant. The isolated converter test provides the measured 0.856–1.018 ms component range. Representative integrated traffic and sustained board operation form the next measurement layer beyond this stage-level model.

9.7. Capacity Bound and Scene Dependence

The 1000-match ceiling is the compile-time capacity bound set by Top-K and the geometry buffers. Actual work depends on scene texture, FIFO admission, bucket capacity, descriptor thresholding, the large-displacement indicator, and pruning. Geometric usefulness additionally depends on correspondence quality and translational parallax, as specified by the operating domain in Section 14.

10. Validation and Results

10.1. Test Methodology

The evaluation combines a three-frame RTL diagnostic using calibrated camera imagery, a 108-pair corpus with saved correspondences and XSim outputs from 42 source identifiers, module-level SVD and triangulation audits, and complete VCU118 implementation results. The corpus supports the displacement, output-count, divider-limit, and fixed-point-software/OpenCV analyses reported below.

The 108-pair corpus targets the architecture's nearby-frame operating envelope: 36 low-, 36 medium-, and 36 high-displacement cases with source-frame gaps of 1–25, loaded into adjacent test slots. It supports repeatable displacement, output-count, divider-limit, and numerical-agreement analyses across the selected conditions. Complementary public-sequence evaluation with annotated camera motion, depth, dynamic content, and inlier ground truth is identified in Section 13.

Table 8 summarizes the validation corpus and the role of each component.

Table 8. Summary of the validation evidence.

Corpus component	Coverage	Validation role
Three-frame RTL diagnostic	Identical-image control followed by one cross-image pair; 940 candidate output rows	Exercises the streaming, matching, and candidate-record path with calibrated camera imagery.
Selected-pair evaluation corpus	108 pairs, 42 source identifiers, 36 pairs per displacement regime, frame gaps 1–25	Supports manifest, count, range, displacement, and divider-limit analyses.
Corpus correspondences and XSim CSVs	100,301 saved correspondences and 100,301 emitted CSV rows; equality holds for every pair	Establishes file completeness and per-pair output cardinality.
Top-K cadence test	30,443 candidates, 43,317 sort clocks, maximum FIFO occupancy 41	No candidates are dropped in the tested trace.
SVD numerical audits	90 temporal-envelope XSim instances and 82 stress instances	All 72 scored nondegenerate temporal instances pass, including 24/24 forward-motion instances; planar/homography stress passes 36/36, while deliberately ill-conditioned stress defines the tested boundary.
Implemented triangulation audit	Exact $W = 12$ RTL; 60 controlled numerical cases; 225-case output/cycle test	The exact implemented source completes every case in 111 solver clocks and passes 40/42 scored cases; the two exceptions are low-angle, ill-conditioned cases. The divider-width test preserves the expected outputs and cycle behavior.
VCU118 implementation	Fully routed, timing-clean, bitstream generated	Establishes complete physical implementation on the target FPGA.

10.2. Stimulus Generation

Each test sequence consists of undistorted 1920×1080 frames converted to hexadecimal stimulus files. When the VHDL testbench is run, these files are fed pixel-by-pixel into the pipeline with HDMI active-pixel, blanking, and VSYNC timing, thereby exercising the VSYNC-triggered bank swap, heap-sort schedule, and geometry-pipeline initiation for that stimulus.

10.3. Multi-Frame Simulation

The supplied validation testbench follows a three-frame simulation template:

- **Frames 0 and 1:** an identical-image pair from the selected triplet's reference viewpoint. This repeatability control exercises the streaming front end and matcher and produces $\sim$999 matches with near-zero Hamming distances. The cross-image stimulus below supplies the nonzero-baseline geometry case.
- **Frame 2:** a second selected viewpoint with baseline separation from the reference frame. This cross-image pair provides parallax for meaningful geometric computation and triangulation.

The labels "Frame 0", "Frame 1", and "Frame 2" denote positions within one three-frame test instance. The 108 selected pairs use source-frame gaps of 1–25 and are loaded into adjacent test slots. A visual audit of one retained frame per source identifier classifies 3 sources as indoor, 4 as semi-covered balcony or parking scenes, and 35 as outdoor; the corpus is therefore dominated by outdoor daylight scenes while retaining ordinary indoor and semi-covered examples. Scene class and pixel-displacement groups provide the descriptive categories for this corpus.

10.4. Verification Criteria

The output path is verified for deterministic repeatability and one-for-one correspondence-count integrity, while the controlled SVD and triangulation audits quantify the geometry stages against float64 references. As with pairwise monocular SfM generally, the recovered structure is defined up to an unknown scale factor [1].

10.5. Focused RTL Numerical Audits

Vivado 2026.1 XSim audits isolate the fixed-point SVD and triangulation solvers and compare raw RTL outputs with a float64 reference computed from the *same quantized inputs*. The tests complete every case and verify the expected handshaking behavior.

Standalone SVD result.

Vivado 2026.1 XSim completed 90 deterministic temporal-envelope instances: 72 scored nondegenerate instances generated from known camera motion and correspondence geometry at selected-view gaps of 1, 10, and 15 frames, plus 18 diagnostic controls. OpenCV 4.10 `SVDecomp` in float64 received the identical signed-Q20.12 matrices as the numerical reference [37]. Failure was predeclared as a relative reconstruction or singular-spectrum error above 0.05, a U or V orthogonality Frobenius error above 0.05, or a sign-invariant left-null-axis error above 5°. All 72 scored instances pass every criterion, including all 24 forward-motion instances. Across those cases, the maxima are 0.022982 for relative reconstruction error, 0.001094 for U orthogonality error, 0.001033 for V orthogonality error, 0.022684 for relative singular-spectrum error, and 0.0360° for left-null-axis error.

A separate 82-instance stress audit uses the same SVD implementation and OpenCV reference: 36 scored planar/homography-dominant matrices, 36 scored deliberately ill-conditioned matrices, and 10 unscored boundary diagnostics with potentially non-unique null subspaces. All 36 planar/homography-dominant instances pass, including all 12 forward-motion instances. The 36 deliberately ill-conditioned instances each exceed at least one predeclared criterion and thereby delineate the tested numerical boundary. Stronger conditioning detection and refinement are natural extensions of this characterized operating envelope.

Triangulation solver.

Vivado 2026.1 XSim evaluated the exact implemented $W = 12$ triangulation source on 60 controlled cases: 42 full-rank scored cases, 12 output-boundary controls, and 6 invalid/degenerate controls. The float64 reference solves the inhomogeneous least-squares system built from the same Q20.12 projection matrices and correspondences. For a scored case, the predeclared coordinate-error limit is $\max(0.05, 0.05 \max(1, \max|\mathbf{X}_{\mathrm{ref}}|))$; the normalized-residual limit is $\max(0.01, 5r_{\mathrm{ref}} + 0.002)$; emission of a divider-boundary word also constitutes an exceedance. Every case completes in 111 solver clocks. A separate 225-case output/cycle test verifies the implemented divider output width and schedule.

Forty of the 42 scored cases pass all criteria. Across the scored cases, maximum absolute coordinate error has p50/p90/maximum values of 0.000751/0.034714/2.733333 coordinate units; p90 relative coordinate error is 0.002476 and p90 normalized algebraic residual is 0.015981. Both exceptions are low-angle, ill-conditioned cases: the 0.04089°, condition-number 2811 case has maximum coordinate error 2.733333 and normalized residual 0.070711, while the 0.06725°, condition-number 1767 case has maximum coordinate error 0.545810 and normalized residual 0.044072. Three of the 12 boundary controls emit at least one explicit divider-limit word. Together, these results characterize numerical agreement across the tested operating region and the expected sensitivity to weak geometry.

10.6. Degenerate Case Handling

For a same-image pair, the zero-baseline pose is unidentifiable: the epipolar equations admit a family of skew-symmetric solutions rather than a unique near-zero essential matrix. The normalized solver estimate can therefore have diagonal terms close to zero while its off-diagonal terms remain nonzero. The `system_controller` does not infer degeneracy from selected diagonal entries. It classifies an essential matrix as numerically collapsed only when all nine signed raw Q20.12 entries have magnitude below 10; only then does it skip pose recovery and triangulation. A valid near-pure translation with $\mathbf{R} \approx \mathbf{I}$ produces $\mathbf{E} \approx [\mathbf{t}]_\times$, so significant off-diagonal entries allow that case to proceed.

The implemented predicate adds no feature-list pass, controller state, or decision cycle. Applying it to the raw-Q20.12 numerical-audit vectors rejected none of the 72 eligible temporal-envelope matrices, including all 24 forward-motion matrices, and none of the 36 planar/homography-dominant matrices; the exact-zero control was rejected. This is a numerical-collapse guard rather than a general two-view degeneracy detector. A future deployment-oriented gate can additionally combine the spatial distribution and support of retained correspondences, cheirality support, triangulation angle, and matrix conditioning.

10.7. Reference-Software Diagnostic and Evaluation-Corpus Audit

OpenCV 4.10 is used as an independent float64 numerical reference on saved pixel-coordinate correspondences. The reference path invokes `findFundamentalMat` in `FM_8POINT` mode to estimate $\mathbf{F}_{\mathrm{cv}}$ and applies calibration as $\mathbf{E}_{\mathrm{cv}} = \mathbf{K}^T \mathbf{F}_{\mathrm{cv}} \mathbf{K}$. Because the corpus has no ground-truth trajectory or metric depth, the reported comparison measures numerical agreement rather than absolute pose, depth, or drift accuracy.

The 108-pair comparison reports the sign-invariant translation-axis difference between the fixed-point software model and OpenCV 4.10 on the same saved correspondences. Low-, medium-, and high-displacement medians are 2.5°, 2.5°, and 1.6°; the high-displacement IQR is 1.1°–3.0° and p90 is 6.7°. The association with pixel displacement is weak (Pearson $r = -0.126$; Spearman $\rho = -0.197$). Pixel displacement is used as an image-space proxy because a metric physical baseline was not recorded.

Figure 4 presents an OpenCV-only diagnostic on one saved correspondence set and shows the sensitivity of positive-depth support to pixel-displacement filtering.

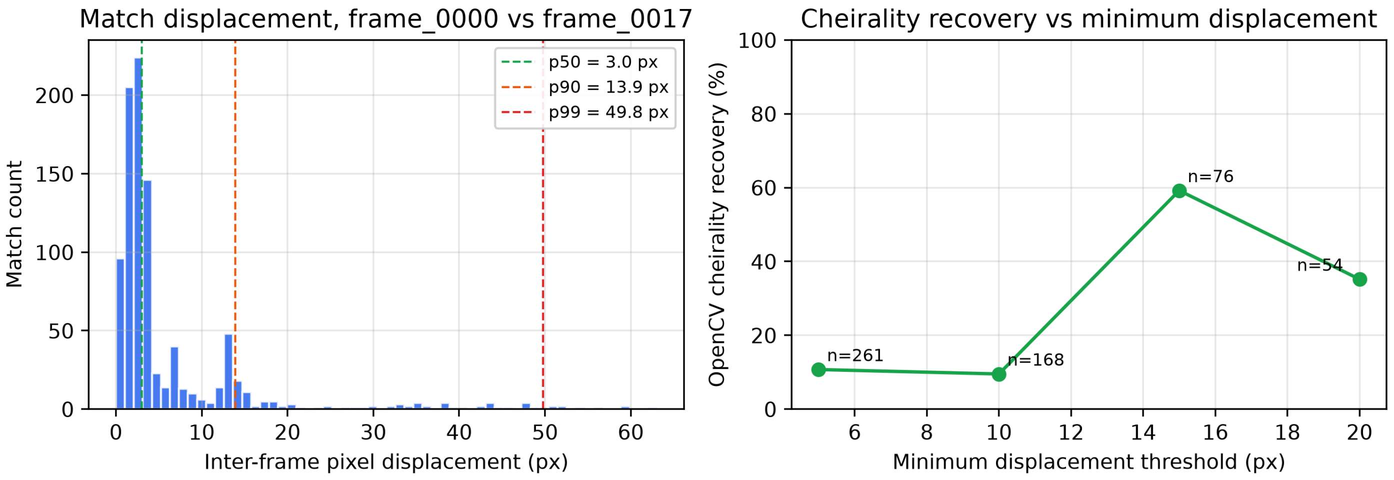

Figure 4. Constrained OpenCV diagnostic for one saved low-displacement correspondence set (source frames 0 and 17; 940 saved matches). Left: the horizontal axis is Euclidean inter-frame displacement in pixels (px), the vertical axis is match count, and blue bars form the histogram; dashed green, orange, and red lines mark p50 (50th percentile/median, 3.0 px), p90 (90th percentile, 13.9 px), and p99 (99th percentile, 49.8 px). Right: the horizontal axis is the minimum retained-displacement threshold in px and the vertical axis is the OpenCV (CV) positive-depth/cheirality fraction in percent; the green line and circles give that fraction and each n label gives the retained match count.

The machine-readable corpus manifest records pair identifier, source identifier, source-frame indices and gap, displacement regime, median saved-correspondence displacement, saved correspondence count, emitted XSim row count, large-displacement and bypass status, exact divider-limit-word counts, and the software-reference translation-axis difference. The retained field names `q8_24_saturated_*` denote equality with those explicit divider limits rather than generic Q8.24 saturation. Table 9 summarizes the verified descriptive fields. A limit hit is recorded when any emitted coordinate equals raw word `0x3FFFFFFF` ($+64 - 2^{-24}$) or `0xC0000000` (-64), the explicit quotient limits in the triangulator's divider.

Table 9. Offline audit of the 108-pair evaluation corpus. Displacement is the median Euclidean image-plane displacement in pixels (px) over saved correspondences. "Clamp hit" means that at least one emitted X, Y, or Z word equals the divider limit `0x3FFFFFFF` ($+64 - 2^{-24}$) or `0xC0000000` (-64).

Regime	Pairs	Median displacement (px)	Median emitted points	Emitted-point range	Pooled clamp-hit rate
Low displacement	36	3.162	963	739–993	28.987%
Medium displacement	36	10.630	937	729–993	15.750%
High displacement	36	24.758	934	797–982	6.882%

Across all 108 pairs, the manifest contains 100,301 saved correspondences and 100,301 emitted XSim rows, with equality for every pair. At least one divider-limit word occurs in 17,359 emitted points (17.307% pooled).

Within the selected corpus, the low-displacement regime spans 2.236–5.000 px, 739–993 saved correspondences, and source-frame gaps of 1–5; the corresponding medians are 3.162 px, 963 correspondences, and 2 frames. The medium regime spans 7.280–14.213 px, 729–993 correspondences, and gaps of 2–8, with medians 10.630 px, 937, and 5. The high regime spans 19.416–33.287 px, 797–982 correspondences, and gaps of 5–25, with medians

24.758 px, 934, and 12. These ranges describe the selected files and are not inferred physical baselines or population coverage.

Figures 5 and 6 show per-pair output count, divider-limit rate, and software-reference translation-axis difference.

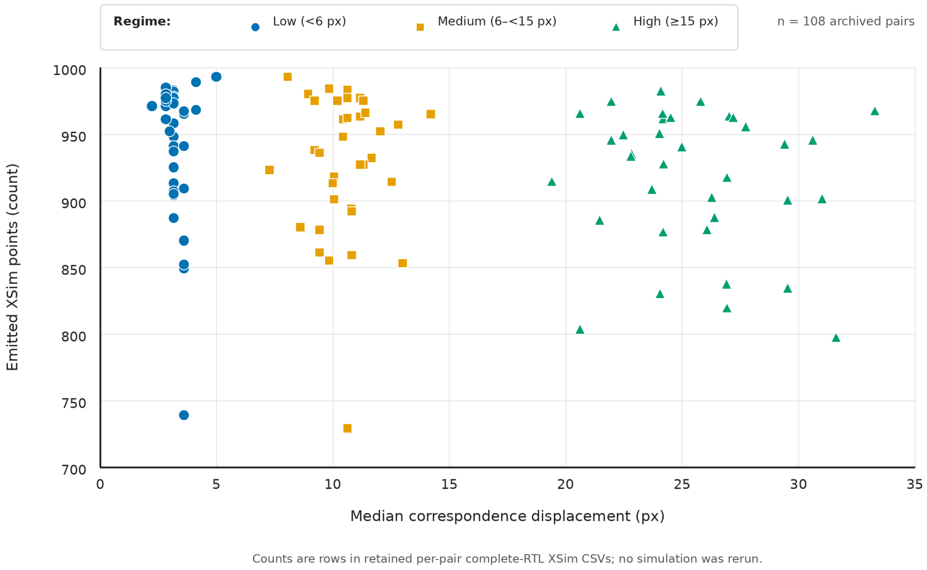

Figure 5. Displacement versus emitted-output count for the 108-pair corpus. The horizontal axis is median Euclidean displacement over saved correspondences in pixels (px); the vertical axis is the number of rows in the per-pair Vivado XSim comma-separated-value (CSV) file. Blue circles denote the low-displacement regime ($<$ 6 px), orange squares the medium regime (6 to $<$ 15 px), and green triangles the high regime ($\geq$ 15 px). Here $r = -0.202$ is Pearson's linear correlation coefficient and $\rho = -0.295$ is Spearman's rank correlation coefficient. Emitted rows equal saved correspondences for every pair.

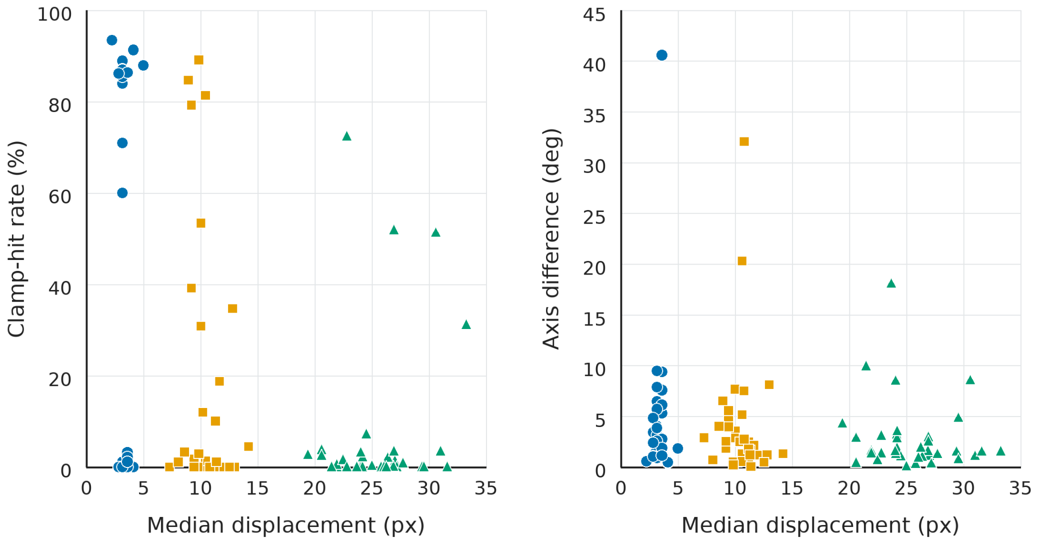

(a) **(b)**

Figure 6. Per-pair diagnostics for the 108-pair corpus. In both panels, the horizontal axis is median Euclidean displacement over saved correspondences in pixels (px); blue circles, orange squares, and green triangles denote the low- (< 6 px), medium- (6 to < 15 px), and high-displacement (≥ 15 px) groups. **(a)** The vertical axis is the percentage of emitted points for which any signed 32-bit Q8.24 X, Y, or Z word (24 fractional bits) equals a divider limit, $+64 - 2^{-24}$ or -64; Pearson $r = -0.263$ and Spearman $\rho = -0.057$. **(b)** The vertical axis is the sign-invariant translation-axis difference in degrees between the fixed-point software model and OpenCV 4.10 on the same saved correspondences; Pearson $r = -0.126$ and Spearman $\rho = -0.197$. Panel (b) reports numerical agreement between the two software estimators.

10.8. Controlled Incorrect-Match Sensitivity Test

Because the corpus does not include ground-truth inlier labels, robustness was tested by controlled coordinate derangement. Within each low-, medium-, and high-displacement regime, the 36 pairs were ordered by median displacement and the nearest-rank 25th, 50th, and 75th percentile entries were selected, giving nine pairs. For each pair, exactly round(qN) second-view coordinates were reassigned at nominal corruption rates $q \in \{0.10, 0.20, 0.30, 0.40\}$. The random mode sampled slots uniformly; the spatially clustered mode chose a seeded first-view anchor and corrupted the nearest slots in image-size-normalized distance. Three fixed seeds (20260822, 20260823, and 20260824) were used. The derangement preserves each coordinate marginal and assigns a different second-view coordinate to every selected slot, although a reassigned pair can occasionally remain geometrically plausible.

The float32 two-pass software estimator was evaluated at five thresholds: 0.05, 0.075, 0.10, 0.125, and 0.15. OpenCV 4.10 `findEssentialMat` RANSAC used probability 0.999, a 1.0-pixel threshold, and at most 1000 iterations. For every pair, mode, rate, and seed, both estimators received byte-identical pixel-coordinate arrays. The complete protocol comprised 225 input variants, 1125 proposed-estimator threshold evaluations, and 225 RANSAC evaluations (1350 result rows).

Failure rules were fixed before execution. A trial failed if the final matrix was nonfinite or fewer than eight correspondences were retained, if clean-correspondence retention fell below 0.50 or retained-set precision fell below 0.90, or if standardized decomposition failed, rotation drift exceeded 5°, or sign-invariant translation-axis drift exceeded 10° relative to the same estimator's uncontaminated result. A pair reached breakdown at the smallest rate for which at least two of three seeds failed; aggregate breakdown was the smallest rate at which at least five of nine pairs reached that rule.

Table 10. Controlled estimator-only incorrect-match sensitivity. Rates are nominal coordinate-derangement fractions; they are not annotated dynamic-object fractions. The proposed result uses the reported $\tau = 0.10$.

Estimator	Random aggregate breakdown	Spatial-cluster aggregate breakdown
Two-pass float32 software estimator ($\tau = 0.10$)	10%	10%
OpenCV 4.10 RANSAC	20%	20%

Across the nine no-injection correspondence sets, all 45 pair–threshold evaluations completed without predeclared failure and retained every correspondence. At $\tau = 0.10$ and nominal 10% random corruption, 25 of 27 proposed-estimator trials failed and all nine pairs met the pair-breakdown rule. Under nominal 10% clustered corruption, 21 of 27 trials failed and seven of nine pairs broke down; the remaining two broke down at 20%. At 20% and every larger tested rate, all 27 proposed-estimator trials failed in both modes. At 10% random corruption, the threshold sweep changed the failure fraction by only 3.7 percentage points: 88.9% for $\tau = 0.05$ and 0.075 and 92.6% for $\tau = 0.10$, 0.125, and 0.15. The clustered fraction was 77.8% for every tested threshold. These results support 0.10 as a stable central setting within the evaluated neighborhood and empirically delineate the intended high-inlier operating domain. Calibrated public sequences can extend this envelope to dynamic and illumination-varying conditions.

This controlled saved-correspondence experiment supports the stated high-inlier operating restriction. Evaluation on calibrated dynamic and illumination-varying public sequences remains future work.

10.9. Performance Analysis

The following subsections report feature extraction (Section 10.10), matching (Section 10.11), and the geometric back end (Section 10.12), followed by the timing model (Section 10.13). Post-route resources, timing, and power are reported in Section 10.14.

10.10. Feature Extraction and Selection Performance

FAST-12 candidate counts per frame are scene-dependent (bounded above by the number of active pixels and below by zero for a textureless frame). The tournament min-heap retains up to 1000 of the strongest candidates admitted through its burst-decoupling FIFO, with the selected count determined by scene content and any FIFO-full drops. In the functional RTL schedule, feature detection accepts 1 pixel per clock without input stalls; overflow is handled by dropping a candidate rather than back-pressuring the pixel stream. The inherited 256-bit BRIEF descriptors are computed at pixel rate without rotation compensation and are stored alongside each feature's coordinates and Harris score in the Top-K pool entry.

10.11. Matching Performance

Simulation results provide the following descriptive matcher observations:

- **Same-viewpoint test:** the three-frame diagnostic contains 999 matches between identical frames (out of 1000 Top-K features). Their near-zero Hamming distances provide a self-consistency check of the detection–description–matching chain.
- **Cross-viewpoint matching:** the diagnostic contains 940 matches for frame_0000 vs frame_0017. This is a scene-dependent observation rather than a pipeline cap; the

hardware is sized for the **1000-feature Top-K ceiling**, which is carried through the throughput accounting in Section 10.13.
- **Search efficiency:** logged examples report $\sim$50–100 candidate comparisons per query feature, compared with the 1000-comparison brute-force ceiling.
- **Bucket overflow:** one logged stimulus reports approximately 85 dropped features per frame, representing $<$9% of the 1000-feature ceiling.

10.12. Geometric Pipeline Performance

- **Algebraic pruning:** applies the configured scale-normalized threshold deterministically to every match. The controlled incorrect-match experiment in Section 10.8 characterizes its operating envelope.
- **Two-pass solver:** the re-solve pass operates on the retained rows of an overdetermined system with at most 1000 rows $\times$ 9 columns (940 in the three-frame diagnostic).
- **Pose recovery:** the RTL constructs four SVD-derived $(\mathbf{R}, \mathbf{t})$ candidates and applies its division-free depth-sign test to match-list entry 0. It selects the first passing candidate in fixed priority order and falls back to the first candidate if none passes. With an immediate SVD response, the pose-controller schedule is 20 clocks.
- **Triangulation:** emits up to the configured match ceiling in signed Q8.24 form. The exact implemented $W = 12$ source completes all 60 controlled cases in 111 solver clocks and passes 40 of 42 scored cases; the two exceptions have ray angles below 0.07°. A 225-case output/cycle test additionally verifies the implemented divider output and cycle behavior.

10.13. Throughput Analysis

Table 11 presents an analytical geometry-back-end subtotal at the **1000-match design ceiling**. Exact VHDL schedule counts are combined with approximately 258,000 wrapper polling events per solver call and a 280,000-cycle loading/control/memory/AXI allowance. Displayed times evaluate VHDL counts at 148.5 MHz and solver polling events at the 49.5 MHz kernel period.

Table 11. Nominal geometry-back-end subtotal at the 1000-match design ceiling. Exact VHDL schedule counts and explicitly identified model assumptions are evaluated at the intended clock periods. Active-raster time, Top-K sorting, the 3000-cycle post-sort interval, matching/preload, representative integrated CDC/arbitration service beyond the isolated converter result, board I/O, point reception, and a worst-case matcher bound are not included.

**Stage**	**Cycles**	**Projected time at intended clock**	**Bottleneck**
Feature detection (active image)	2,073,600 accepted-pixel cycles	13.96 ms of active pixels	Complete raster is 2,475,000 link clocks; outside subtotal
Top-K selection	overlapped	N/A	Within detection
Top-K heap-sort (one trace)	43,317 `pix_clk`	0.292 ms at 148.5 MHz	Post-VSYNC; trace-specific; outside subtotal
Spatial-bucket matching	scene-dependent	Not bounded	Post-VSYNC; workload dependent
Eight-point solver (Pass 1)	∼258,000 polling events	**nominal 5.21 ms**	One-event/one-kernel-period model assumption
Algebraic pruning	∼1008 `pix_clk` start-to-done	0.0068 ms at 148.5 MHz	Approximately 1006 feed/drain cycles plus FSM boundaries
Eight-point solver (Pass 2)	∼258,000 polling events	**nominal 5.21 ms**	Same model assumption as Pass 1
SVD + pose recovery	conservative 531 `pix_clk`	0.004 ms at 148.5 MHz	Fixed-point SVD range 175–451; pose schedule 20 clocks with immediate response
Triangulation (1000 pts, W=12)	120,000 `pix_clk`	0.808 ms at 148.5 MHz	111 solver + 9 controller clocks per point
Loading + wrapper + memory + AXI allowance	∼280,000 `pix_clk`	nominal 1.886 ms	Analytical model allowance
**Nominal geometry-back-end subtotal**	—	∼**13.13 ms**	Analytical subtotal under the stated assumptions
**Frame period at 60 fps**	2,475,000	16.67 ms	N/A

Under those assumptions, the chosen arithmetic is

$$T_{\text{nom}} = 2\left(\frac{258000}{49.5 \times 10^{6}}\right) + \frac{1008 + 531 + 120000 + 280000}{148.5 \times 10^{6}}$$
$$= 13.128 \text{ ms},$$

rounded to 13.13 ms. The two nominal solver terms contribute 10.424 ms, the loading/control/memory/AXI allowance 1.886 ms, triangulation 0.808 ms, pruning 0.0068 ms, and the conservative SVD-plus-pose term 0.0036 ms. The active raster, Top-K sorting, fixed post-sort interval, matcher/preload handoff, representative integrated CDC/arbitration service beyond the isolated converter result, board I/O, point-record reception, and a worst-case matching bound are excluded. The subtotal is therefore not subtracted from the frame period and no complete-system headroom is reported.

Compute capacity and geometric usefulness are distinct. The matcher raises a large-displacement indicator when at least one accepted match exceeds 60 pixels in either image axis. The signal is intentionally treated only as image-motion evidence; translational observability belongs to the separate correspondence-support, cheirality, parallax, triangulation-angle, and conditioning checks identified for deployment.

Figure 7 shows that the two nominal solver terms dominate the approximately 13.13 ms inferred subtotal.

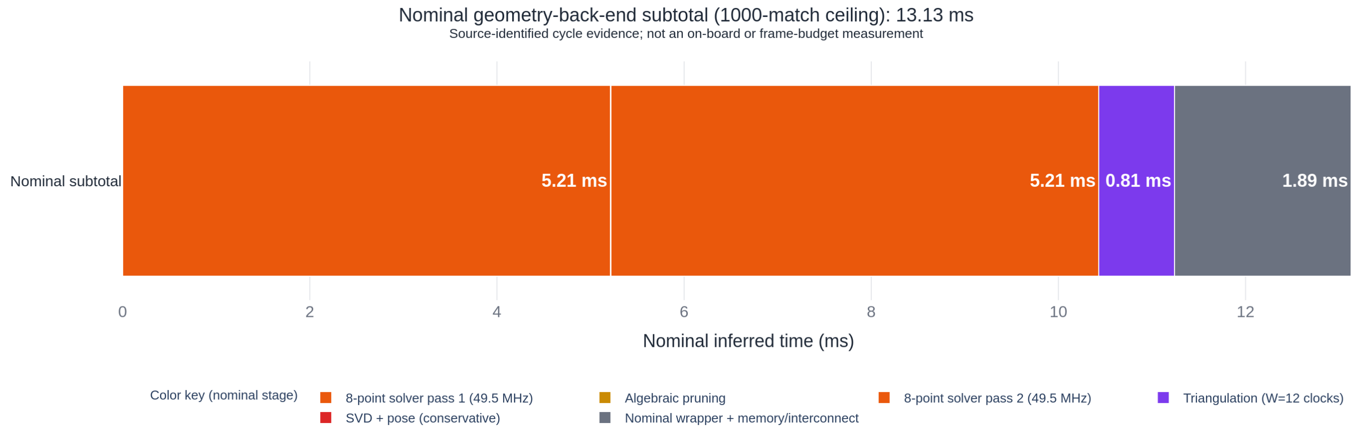

Figure 7. Nominal geometry-back-end subtotal at the 1000-match ceiling (∼13.13 ms). The horizontal axis is nominal inferred time in milliseconds (ms), and the single vertical category is the stacked nominal subtotal. Orange denotes two solver terms obtained by treating each recorded wrapper polling event as one 49.5 MHz kernel period (10.424 ms combined); yellow denotes algebraic pruning; purple denotes triangulation with arithmetic wait count $W = 12$ clock cycles per arithmetic-heavy step (0.808 ms); red denotes singular-value-decomposition (SVD) pose recovery (0.004 ms); and gray denotes the wrapper/interconnect/match-loading allowance (1.886 ms). Exact hardware schedules are evaluated at 148.5 MHz. The active raster, Top-K, fixed post-sort interval, matching/preload, representative integrated clock-domain-crossing (CDC) and arbitration service beyond the isolated converter result, board input/output, point reception, and a worst-case matcher bound are excluded, so this is an inferred planning number rather than an on-board or frame-budget measurement.

10.14. Post-Route Resources, Timing, and Power

Table 12 reports resources from the exact routed VCU118 checkpoint used to generate the final bitstream. The implemented design includes the board I/O wrapper, all programmable-logic pipeline stages, clock conversion and AXI infrastructure, the BRAM-backed Top-K, and the 128 KiB on-chip solver-transaction endpoint.

Table 12. Final post-route resource utilization on the VCU118 XCVU9P using Vivado 2026.1. A Block RAM tile equals one RAMB36 or two RAMB18 blocks.

Resource	Post-route used	XCVU9P capacity	Utilization
CLB LUTs	142,953	1,182,240	12.09%
CLB registers	82,418	2,364,480	3.49%
Block RAM tiles	711.5	2160	32.94%
RAMB36 / RAMB18	684 / 55	2160 / 4320	31.67% / 1.27%
UltraRAM	0	960	0%
DSP slices	1293	6840	18.90%
Bonded I/O buffers (IOBs)	149	832	17.91%

The final route contains 292,748 routable nets, all fully routed, with zero routing errors. Static timing analysis reports WNS = +0.031 ns, TNS = 0, WHS = +0.010 ns, THS = 0, and worst pulse-width slack = +1.347 ns, with zero failing setup, hold, or pulse-width endpoints. The design uses a 148.5 MHz pixel/back-end clock and a 49.5 MHz HLS-kernel clock. Source–destination-specific multicycle setup/hold constraints are applied only to arithmetic paths whose RTL schedules intentionally hold the source and delay capture; the triangulator feedback path uses an explicit 12-clock wait counter matched by 12-cycle setup and 11-cycle hold analysis. Other synchronous paths retain their native clock requirements. The reported slack is the final sign-off result for this implemented, schedule-matched constraint set.

Vivado Power Analyzer was run twice on that same routed checkpoint using the methodology described in [36]. The vectorless analysis estimates 4.534 W total on-chip power (2.030 W dynamic and 2.504 W device-static) and a junction temperature of 27.5°C. The activity-based analysis uses a 70-ms SAIF trace captured from the retained pipeline simulation and estimates 4.296 W total (1.796 W dynamic and 2.501 W device-static; the 0.001 W displayed-sum difference is rounding) and 27.3°C. Both reports have *Low* overall confidence. For the activity-based result, dynamic components include 0.298 W for signals, 0.312 W for CLB logic, 0.383 W for Block RAM, 0.296 W for DSPs, 0.260 W for clocks, 0.194 W for MMCMs, and 0.051 W for I/O (small categories and rounding account for the remaining difference). Both analyses assume a typical process, 25°C ambient temperature, 250 LFM airflow, a medium-profile heat sink, and a medium 10×10-inch board. The activity-based run applies the compatible captured annotations to the corresponding routed logic and uses Vivado's standard activity propagation for the remaining design. The two methods differ by 0.238 W and span 4.296–4.534 W under two activity assumptions. They are reported as post-route tool estimates rather than physical board-power measurements.

The resulting 80,159,324-byte bitstream has SHA-256 `1257def2115839cee1ea6030b8d4954e6655baa3d3849de3f6818bcb2a208e13`. It was generated after a routed DRC report with zero Error/Fatal findings; the report's warnings and advisories are retained in the supplementary evidence.

For the smaller `xczu19eg-ffvc1760-2-e`, logic optimization of the full register-backed design required 532,817 of 522,720 CLB LUTs: a measured gap of 10,097 LUTs, or 1.93% (about 2%). This result narrows the remaining target for small-device closure. Applying the final Block-RAM-backed Top-K organization and completing exact-part placement, routing, timing, and bitstream generation on XCZU19EG form the corresponding future implementation study.

11. Hardware-Oriented Algorithmic Trade-offs

The architecture specializes a standard software SfM pipeline (e.g., COLMAP [2], ORB-SLAM3 [3]) for deterministic programmable-logic execution. This section explains the resulting algorithmic substitutions, pairwise scope, and hardware rationale.

11.1. Algebraic Pruning Instead of RANSAC

Standard approach: Random Sample Consensus [20] iteratively selects random minimal subsets (e.g., 8 matches), fits a fundamental matrix, counts inliers, and retains the best model after $N = 500$–2000 iterations.

Design choice: a two-pass deterministic scheme. (1) Solve from the over-determined 1000×9 design matrix using the Gram-matrix/inverse-power kernel. (2) Apply single-pass algebraic-error pruning (pipelined, 6 stages, one decision per clock). (3) Re-solve using retained matches.

Justification: the initial over-determined solve provides a usable least-squares estimate when the selected pair has a high inlier ratio. Reusing the nominal 5.21 ms full-data solver term for 500 hypotheses would project to approximately 2.6 s per pair before integration overhead. The two-pass scheme is therefore a throughput-oriented choice for the intended predominantly static, high-inlier selected-pair regime.

Bounded sensitivity experiment: we evaluated the float32 two-pass software estimator on nine displacement-stratified saved correspondence sets. Random and spatially clustered coordinate derangements were injected at nominal rates of 10%, 20%, 30%, and 40% with three fixed seeds. Under a predeclared composite failure criterion (finite/support checks, clean-match retention and precision, and rotation/translation drift relative to each estimator's uncontaminated result), the proposed estimator at $\tau = 0.10$ reached aggregate

breakdown at nominal 10% corruption for both modes, while OpenCV RANSAC reached aggregate breakdown at nominal 20%. Because a coordinate derangement can occasionally remain geometrically plausible, these are nominal corruption rates.

Fixed-point RTL calibration set the coefficient: 0.05 produced an eight-LSB Q20.12 cutoff, below matcher-retained cross-frame residuals of about 11 LSBs from quantization and rank-2 projection; 0.10 raised it to about 16 LSBs. A separate float32 sweep of five values from 0.05 to 0.15 retained every correspondence in all 45 no-injection evaluations. At nominal 10% corruption, failure fractions differed by only 3.7 points for random corruption and were identical for clustered corruption. Thus 0.10 is the implementation-calibrated central tolerance above the observed numerical floor and is locally insensitive across the tested sweep; independently labelled correspondences can support application-specific refinement.

Operating scope: the estimator is designed for predominantly static, high-inlier, high-overlap nearby pairs with translational parallax. Broader dynamic, wide-baseline, or low-inlier operation can be addressed by adding robust hypothesis testing and a stronger motion/parallax gate.

11.2. Deterministic Matching Trade-off

Standard approach: Lowe's ratio test [23] rejects ambiguous matches by requiring $d_1/d_2 < 0.75$, where d_1 and d_2 are the best and second-best Hamming distances.

Our replacement: a spatial bucket constraint (search limited to the current bucket and its eight neighbors) combined with an absolute Hamming threshold ($\leq$106/256 bits).

Justification: the spatial constraint reduces the candidate set and suppresses distant false matches while avoiding a second-best-distance search. The combination of spatial restriction, the absolute Hamming threshold, and epipolar pruning is optimized for the stated high-overlap, high-inlier operating domain.

11.3. Pairwise Scope and Host-Side Refinement

Standard approach: bundle adjustment (BA) [33] jointly refines all camera poses and 3D points by minimizing reprojection error across all views, a large-scale non-linear least-squares problem.

Our approach: selected-pair relative-pose estimation ($\mathbf{R}, \mathbf{t}$ for one pair), with trajectory accumulation and global refinement assigned to an optional host-side extension.

Justification: BA requires iterative Levenberg–Marquardt optimization over many parameters and sparse Jacobian computation, which is outside this fixed pairwise datapath. A future hybrid system could pass pairwise poses and points from PL to a host-side sliding-window optimizer through a buffered transport interface, but its rate and convergence would require separate implementation and validation.

11.4. Pairwise Scope Relative to Loop Closure

Loop closure detects when the camera revisits a previously observed location, enabling drift correction and global map consistency. It requires a visual vocabulary (Bag of Words) for place recognition and matching against *all* stored keyframes, followed by pose graph optimization. The present datapath supplies selected-pair motion and feature records that a future keyframe database and pose-graph module could consume; retrieval, keyframe storage, and graph optimization constitute a complementary research contribution.

11.5. Monocular Scale Convention

Monocular SfM recovers 3D structure only up to an unknown scale factor [1]. The translation vector $\mathbf{t}$ is recovered with unit norm, and all 3D points reside in an arbitrary-scale coordinate system. This is an inherent property of monocular vision, not a limitation

of the hardware implementation. Absolute scale recovery requires additional sensors (stereo baseline, IMU) or known scene dimensions.

11.6. Single-Scale Feature Front End

Standard ORB builds an 8-level image pyramid for scale invariance [24]. The present front end operates at native 1920×1080 scale and is optimized for selected nearby pairs with bounded apparent scale change. Wider-baseline matching and loop closure can extend this architecture with additional pyramid levels, row buffers, and a scale-aware matching policy.

11.7. Summary of Trade-offs

Table 13 consolidates the principal algorithmic substitutions and their hardware rationale.

Table 13. Summary of algorithmic trade-offs between a standard software SfM pipeline and our FPGA implementation.

Standard SfM Stage	Our Approach	Justification
RANSAC	Two-pass algebraic pruning	Deterministic and pipelineable for the reported high-inlier video regime.
Lowe's ratio test	Spatial bucket + Hamming threshold	Spatial restriction and thresholding provide a bounded deterministic matcher.
Bundle adjustment	Per-pair $(\mathbf{R}, \mathbf{t})$ in PL	Host-side sliding-window refinement is a complementary extension.
Loop closure	Pairwise feature and pose outputs	Supplies inputs for a future keyframe database and pose-graph module.
Scale estimation	Unit-norm translation	Inherent monocular SfM limitation, not hardware-specific.
Image pyramid	Single-scale detection	Optimized for selected nearby pairs; additional levels extend the design toward wider baselines.

12. Discussion

12.1. Comparison with Software Systems

Table 14 contrasts our FPGA pipeline with representative software SfM systems.

Table 14. Comparison against representative software SfM systems.

System	Platform	Scope	Resolution	Geometry Execution	Evidence reported
COLMAP [2]	Desktop CPU/GPU	Incremental SfM with bundle adjustment	Arbitrary	Software	Offline batch
ORB-SLAM3 [3]	CPU	Visual and visual–inertial SLAM	Arbitrary	Software	Real time; platform dependent
eSLAM [15]	Zynq FPGA+CPU	ORB-SLAM acceleration	N/A	Partial hardware acceleration	Application dependent
Komorkiewicz et al. [17]	Zynq FPGA+ARM	SfM pipeline	720×576	ARM software for RANSAC/SVD	50 fps
This work	VCU118 / XCVU9P	Pairwise monocular SfM	1920×1080	Complete listed chain in PL	Stage-complete PL mapping; routed timing closure; bitstream generation; controlled numerical evaluation†

† The 13.13 ms geometry-back-end subtotal combines exact VHDL schedule counts with explicit functional-model assumptions. It is reported separately from end-to-end latency and sustained board throughput; full-device post-route timing is the independent physical result.

Vivado Power Analyzer gives two Low-confidence post-route estimates for the routed VCU118 design [36]: 4.534 W under vectorless activity assumptions and 4.296 W with partial SAIF annotation. The principal result of Table 14 is architectural and physical: every geometric stage is assigned to programmable logic, the board design is routed and timing-clean, and a bitstream is generated. Section 10.14 reports the complete activity-coverage assumptions.

12.2. Fixed-Point Precision

The controlled audits in Section 10.5 characterize the implemented numerical operating envelope: the fixed-point SVD passes all 72 scored nondegenerate temporal-envelope cases and all 36 planar stress cases, and the triangulator passes 40 of 42 scored cases, with two low-angle, ill-conditioned cases defining its measured boundary. The `sfixed(19,-12)` interface format is signed Q20.12 with representation step $2^{-12} = 0.000244$, while `sfixed(3,-28)` represents reciprocal focal lengths. The eight-point HLS kernel operates in IEEE 754 float32, and the pure-VHDL 3×3 SVD and triangulation elimination use signed 32-bit Q8.24 internally; "24" is the fractional-bit count, not the word length. Forming normal equations can approximately square the input condition number, and the fixed total word length has finite range and precision, so overflow, underflow, divider-output limiting, and weak-parallax sensitivity remain possible outside the characterized envelope. The 36 deliberately ill-conditioned SVD stress cases and the corpus occurrences of the divider's explicit -64 and $+64 - 2^{-24}$ quotient limits document those boundaries.

12.3. Position in the Literature

Table 2 gives a bounded comparison with representative FPGA systems. To the authors' knowledge, and among the directly comparable systems surveyed there, this is the first reported stage-complete programmable-logic mapping of the full pairwise SfM chain. FAST-12, Harris–Stephens, and 256-bit unrotated BRIEF use the algorithmic front end of [5] and are connected to the interfaces defined here. The remaining components are the Block-RAM-backed Top-K, spatial-bucket matcher, normalization/A-matrix formatting, HLS solver integration, pruning and re-solve, pure-VHDL SVD, single-correspondence pose selector, triangulation, inter-frame control, and back-end cycle/clock methodology. The complete VCU118 implementation establishes device fit, path-specific timing closure, and bitstream generation for this architecture.

13. Future Work

13.1. Live-Board Characterization and Small-Device Porting

The VCU118 design is routed, timing-clean, and bitstream-generating. Live-board studies can extend this physical result with decoded-video capture, source-synchronous point-record reception, sustained-traffic comparison against stage-level reference data, representative dual-clock AXI/CDC service measurements, broader full-design VCD or SAIF activity, and physical rail-power measurements.

The full XCZU19EG design using a register-backed Top-K exceeded CLB-LUT capacity by 1.93% (about 2%) after logic optimization. The packed 1024×349 Block-RAM pool used by the VCU118 architecture supplies the focused optimization route for a future exact-part XCZU19EG synthesis, placement, routing, timing, and bitstream study.

The characterized numerical envelope motivates stronger conditioning detection and refinement, ground-truth pose-error distributions against displacement and known parallax or baseline on calibrated public sequences, and dynamic and illumination-varying studies. An extended motion gate can combine correspondence distribution and support, cheirality, triangulation angle, and matrix conditioning. Optional buffering or receiver-side flow control can support downstream consumers that cannot accept every asserted point record.

13.2. Bundle Adjustment on a Host or External Processor

A host or external processor could consume the emitted relative poses and point-coordinate records, accumulate them into a sliding-window multi-frame state, and apply lightweight bundle adjustment. Such a hybrid extension requires a buffered transport interface and separate rate and convergence characterization.

13.3. Loop Closure and Multi-Map

With features and descriptors available from the streaming front end, a vocabulary-based place-recognition module could detect revisits and trigger pose-graph optimization on an external host processor. This would extend the pipeline from pairwise SfM toward full visual SLAM; the extension would require separate rate and accuracy validation.

13.4. Multi-Scale Feature Detection

Adding 2–4 pyramid levels would improve robustness to scale changes for wider-baseline applications. Each level requires additional row buffers (BRAM cost linear in width per level) and a scale-aware matching strategy.

13.5. Higher Resolution and Frame Rate

The normalized-coordinate geometry back end is resolution-independent in principle. Extending the complete architecture to 4K60 requires revised row-buffer dimensions,

bucket geometry, memory sizing, and likely a multi-pixel-per-clock front end, followed by functional simulation, synthesis, implementation, and timing validation.

14. Operating Domain and Deployment Scope

The following assumptions define the operating domain for which the pairwise architecture and its deterministic hardware schedule were designed.

1. **Undistorted input video.** The input HDMI stream must be lens-undistorted prior to pipeline entry. Radial and tangential distortion would corrupt feature positions and all downstream geometry. Undistortion may be performed by the video source, a host-side preprocessor, or a dedicated PL module.
2. **Known camera intrinsics.** The camera matrix K (focal lengths f_x, f_y and principal point c_x, c_y) must be known from offline calibration [32] and is hardcoded into the VHDL design. Changing the camera or lens requires re-synthesis; these are not runtime-configurable parameters in the current implementation.
3. **Predominantly static, high-inlier content.** The algebraic pruner is optimized for pairs in which descriptor and spatial gates provide a high initial inlier ratio. Dynamic-object rejection and robust hypothesis search extend the architecture toward moving content, repeated texture, and abrupt illumination change.
4. **Sufficient texture.** FAST feature detection requires textured regions. Textureless surfaces (blank walls, sky, uniform floors) yield few or no features, degrading matching and reconstruction quality.
5. **Selected nearby-pair processing.** The matcher operates on two adjacent internal frame slots and targets smooth bounded motion with sufficient feature overlap inside the 3×3 bucket neighborhood. The selected source-frame gaps of 1–25 provide a range of nearby-pair separations.
6. **Non-negligible translational parallax.** The 60-pixel signal is an image-displacement indicator, while the whole-matrix numerical-collapse guard preserves valid translation with near-zero diagonal entries. Deployment-oriented extensions can combine correspondence support, cheirality support, parallax, triangulation angle, and matrix conditioning to distinguish translation from rotation, moving objects, and outliers.
7. **Bounded feature admission.** Top-K has a compile-time ceiling of 1000, and the 64-entry ingress FIFO drops later candidates when full rather than stalling pixels. Dense raster-localized bursts can therefore introduce spatial admission bias; no pre-Top-K spatial quota is implemented.
8. **Fixed resolution.** The implemented and validated pipeline targets 1920×1080 input with a 60 fps architectural input target. Other resolutions require recomputing row-buffer depths, bucket-grid dimensions, memory capacities, and timing parameters at synthesis time.
9. **Single monocular camera.** The system processes one video stream; replicated front ends can extend the architecture to stereo or multi-camera configurations.
10. **Pairwise scale and trajectory scope.** As with pairwise monocular SfM, translation and reconstruction are up to an unknown scale factor. Bundle adjustment, loop closure, global optimization, trajectory accumulation, drift correction, and absolute-scale sensing are complementary multi-frame extensions.

15. Conclusions

To the authors' knowledge, and within the directly comparable FPGA vision literature surveyed in Table 2, this work is the first reported stage-complete programmable-logic mapping of pairwise monocular SfM. Starting from a calibrated, pre-undistorted decoded video stream, the architecture implements feature extraction and selection, correspondence

matching, essential-matrix estimation and pruning, SVD-based pose recovery, and triangulation through a source-synchronous point-coordinate output, without processor-side SfM computation.

The complete VCU118/XCVU9P design was linked without black boxes, placed, physically optimized, and fully routed. Vivado 2026.1 routed all 292,748 routable nets without errors, closed setup, hold, and pulse-width timing with WNS +0.031 ns, WHS +0.010 ns, and WPWS +1.347 ns, and generated an 80,159,324-byte bitstream. The routed implementation uses 12.09% of CLB LUTs, 3.49% of CLB registers, 32.94% of Block RAM tiles, and 18.90% of DSPs. Controlled module-level evaluation characterizes the numerical operating domain of the fixed-point SVD and triangulator, and deterministic corpus replay confirms one emitted output row for every saved correspondence. Under the stated cycle assumptions, the geometry-back-end subtotal is 13.13 ms at the 1000-match ceiling.

Together, these results constitute the first reported stage-complete, timing-closed, bitstream-generated realization of pairwise monocular SfM entirely in FPGA programmable logic within the surveyed directly comparable literature. They demonstrate that every listed stage of the complete two-view chain can reside in programmable logic in one routed 1920×1080 design, establishing a reproducible physical reference for future FPGA SfM systems.

Author Contributions: Conceptualization, Panteleimon Stamatakis; investigation, Panteleimon Stamatakis; methodology, Panteleimon Stamatakis; software, Panteleimon Stamatakis; validation, Panteleimon Stamatakis; writing of the original draft, Panteleimon Stamatakis; review and editing, John Vourvoulakis. All authors have read and agreed to the published version of the manuscript.

Funding: This research received no external funding.

Data Availability Statement: The supplementary materials contain the complete selected 108-pair image corpus (216 calibrated 1920×1080 JPEGs), the calibration file, the machine-readable pair manifest, result summaries, analysis scripts, implementation source, source manifests, VCU118 constraints, routed implementation reports, routed-checkpoint identity, and bitstream SHA-256. The exact selected frame pairs used in the reported corpus analysis are included, providing a reproducible basis for the reported validation and subsequent benchmark extensions.

Conflicts of Interest: The authors declare no conflicts of interest.

Abbreviations

The following abbreviations are used in this manuscript:

AXI	Advanced eXtensible Interface
BA	Bundle Adjustment
BRAM	Block Random-Access Memory
BRIEF	Binary Robust Independent Elementary Features
CDRAM	Coordinate Directory RAM (spatial bucket directory)
CPRAM	Crossbar Point RAM (feature descriptors)
DDR	Double Data Rate (memory)
DLT	Direct Linear Transform
DSP	Digital Signal Processing (slice)
FAST	Features from Accelerated Segment Test
FF	Flip-Flop
FPGA	Field-Programmable Gate Array
FSM	Finite State Machine
HDMI	High-Definition Multimedia Interface
HLS	High-Level Synthesis
LUT	Look-Up Table
MAC	Multiply-Accumulate
MCP	Multicycle Path
ORB	Oriented FAST and Rotated BRIEF
PL	Programmable Logic
RANSAC	Random Sample Consensus
SfM	Structure from Motion
SLAM	Simultaneous Localization and Mapping
SoC	System on Chip
SVD	Singular Value Decomposition
Top-K	Top-K Selection (tournament min-heap)
VDMA	Video Direct Memory Access
VHDL	VHSIC Hardware Description Language
VSYNC	Vertical Synchronization

References

1. Hartley, R.; Zisserman, A. *Multiple View Geometry in Computer Vision*, 2nd ed.; Cambridge University Press: Cambridge, UK, 2004; ISBN 0-521-54051-8; https://doi.org/10.1017/CBO9780511811685.
2. Schönberger, J.L.; Frahm, J.-M. Structure-from-Motion Revisited. In Proceedings of the IEEE Conference on Computer Vision and Pattern Recognition (CVPR), Las Vegas, NV, USA, 26 June–1 July 2016; pp. 4104–4113.
3. Campos, C.; Elvira, R.; Gómez Rodríguez, J.J.; Montiel, J.M.M.; Tardós, J.D. ORB-SLAM3: An Accurate Open-Source Library for Visual, Visual-Inertial, and Multimap SLAM. *IEEE Trans. Robot.* **2021**, *37*, 1874–1890.
4. Bradski, G. The OpenCV Library. *Dr. Dobb's J. Softw. Tools* **2000**, *25*, 120, 122–125.
5. Stamatakis, P.; Vourvoulakis, J. High-Throughput ORB Feature Extraction on Zynq SoC for Real-Time Structure-from-Motion Pipelines. *J. Imaging* **2025**, *11*, 178. https://doi.org/10.3390/jimaging11060178.
6. Weberruss, J.; Kleeman, L.; Drummond, T. ORB Feature Extraction and Matching in Hardware. In Proceedings of the Australasian Conference on Robotics and Automation (ACRA), Canberra, Australia, 2–4 December 2015; pp. 61–70.
7. Weberruss, J.; Kleeman, L.; Boland, D.; Drummond, T. FPGA Acceleration of Multilevel ORB Feature Extraction for Computer Vision. In Proceedings of the 27th International Conference on Field Programmable Logic and Applications (FPL), Ghent, Belgium, 4–8 September 2017; pp. 435–442.
8. Fang, W.; Zhang, Y.; Yu, B.; Liu, S. FPGA-Based ORB Feature Extraction for Real-Time Visual SLAM. In Proceedings of the International Conference on Field Programmable Technology (ICFPT), Melbourne, Australia, 11–13 December 2017; pp. 275–278.
9. Wasala, M.; Szolc, H.; Kryjak, T. An Efficient Real-Time FPGA-Based ORB Feature Extraction for an UHD Video Stream for Embedded Visual SLAM. *Electronics* **2022**, *11*, 2259.
10. Belmessaoud, N.M.; Bentoutou, Y.; Chikr El-Mezouar, M. FPGA Implementation of Feature Detection and Matching Using ORB. *Microprocess. Microsyst.* **2022**, *94*, 104666.
11. Huang, B.C.; Zhang, Y.J. A High-Efficiency FPGA-Based ORB Feature Matching System. *J. Circuits Syst. Comput.* **2024**, *33*, 2450028.
12. Fularz, M.; Kraft, M.; Schmidt, A.; Kasiński, A. A High-Performance FPGA-Based Image Feature Detector and Matcher Based on the FAST and BRIEF Algorithms. *Int. J. Adv. Robot. Syst.* **2015**, *12*, 141. https://doi.org/10.5772/61434.

13. Mattoccia, S. Stereo Vision Algorithms for FPGAs. In Proceedings of the IEEE Conference on Computer Vision and Pattern Recognition Workshops (CVPRW), Portland, OR, USA, 23–28 June 2013; pp. 636–641. https://doi.org/10.1109/CVPRW.2013.96.
14. Zhao, J.; Liang, T.; Feng, L.; Ding, W.; Sinha, S.; Zhang, W.; Shen, S. FP-Stereo: Hardware-Efficient Stereo Vision for Embedded Applications. In Proceedings of the 30th International Conference on Field Programmable Logic and Applications (FPL), Gothenburg, Sweden, 31 August–4 September 2020; pp. 269–276. https://doi.org/10.1109/FPL50879.2020.00052.
15. Liu, R.; Yang, J.; Chen, Y.; Zhao, W. eSLAM: An Energy-Efficient Accelerator for Real-Time ORB-SLAM on FPGA Platform. In Proceedings of the 56th ACM/IEEE Design Automation Conference (DAC), Las Vegas, NV, USA, 2–6 June 2019; article 193.
16. Wang, C.; Liu, Y.; Zuo, K.; Tong, J.; Ding, Y.; Ren, P. ac^{2}SLAM: FPGA Accelerated High-Accuracy SLAM with Heapsort and Parallel Keypoint Extractor. In Proceedings of the International Conference on Field-Programmable Technology (ICFPT), Auckland, New Zealand, 6–10 December 2021; pp. 1–9.
17. Komorkiewicz, M.; Kryjak, T.; Chuchacz-Kowalczyk, K.; Skruch, P.; Gorgoń, M. FPGA Based System for Real-Time Structure from Motion Computation. In Proceedings of the Conference on Design and Architectures for Signal and Image Processing (DASIP), Kraków, Poland, 23–25 September 2015; pp. 1–7.
18. Hartley, R.I. In Defense of the Eight-Point Algorithm. *IEEE Trans. Pattern Anal. Mach. Intell.* **1997**, *19*, 580–593.
19. Longuet-Higgins, H.C. A Computer Algorithm for Reconstructing a Scene from Two Projections. *Nature* **1981**, *293*, 133–135.
20. Fischler, M.A.; Bolles, R.C. Random Sample Consensus: A Paradigm for Model Fitting with Applications to Image Analysis and Automated Cartography. *Commun. ACM* **1981**, *24*, 381–395.
21. Rosten, E.; Drummond, T. Machine Learning for High-Speed Corner Detection. In Proceedings of the European Conference on Computer Vision (ECCV); Lecture Notes in Computer Science; Springer: Berlin, Germany, 2006; Volume 3951, pp. 430–443.
22. Harris, C.; Stephens, M. A Combined Corner and Edge Detector. In Proceedings of the 4th Alvey Vision Conference, Manchester, UK, 31 August–2 September 1988; pp. 147–151.
23. Lowe, D.G. Distinctive Image Features from Scale-Invariant Keypoints. *Int. J. Comput. Vis.* **2004**, *60*, 91–110.
24. Rublee, E.; Rabaud, V.; Konolige, K.; Bradski, G. ORB: An Efficient Alternative to SIFT or SURF. In Proceedings of the IEEE International Conference on Computer Vision (ICCV), Barcelona, Spain, 6–13 November 2011; pp. 2564–2571.
25. Huang, J.; Zhou, G.; Zhou, X.; Zhang, R. A New FPGA Architecture of FAST and BRIEF Algorithm for On-Board Corner Detection and Matching. *Sensors* **2018**, *18*, 1014.
26. Fularz, M.; Kraft, M.; Schmidt, A.; Kasiński, A.J. FPGA Implementation of the Robust Essential Matrix Estimation with RANSAC and the 8-Point and the 5-Point Method. In *Facing the Multicore-Challenge II*; Lecture Notes in Computer Science; Springer: Berlin, Germany, 2012; Volume 7174, pp. 60–71. https://doi.org/10.1007/978-3-642-30397-5_6.
27. El Bouazzaoui, I.; Rodríguez Flórez, S.; El Ouardi, A. FPGA Architecture-Based Front-End Processing for SLAM Applications. *J. Real-Time Image Process.* **2025**, *22*, 73. https://doi.org/10.1007/s11554-025-01650-w.
28. Arora, B.; Gottschaldt, P.; Podlubne, A.; Pertuz, S.A.; Göhringer, D. SoC-SLAM: FPGA-Based Hardware/Software Co-Design for Real-Time Visual SLAM Front-End and Back-End Acceleration. In Proceedings of the 28th Euromicro Conference on Digital System Design (DSD), Salerno, Italy, 10–12 September 2025; pp. 549–557. https://doi.org/10.1109/DSD67783.2025.00081.
29. Kim, S.; Hsiao, R.; Nikolić, B.; Demmel, J.; Shao, Y.S. SuperNoVA: Algorithm-Hardware Co-Design for Resource-Aware SLAM. In Proceedings of the 30th ACM International Conference on Architectural Support for Programming Languages and Operating Systems, Volume 1 (ASPLOS), Rotterdam, The Netherlands, 30 March–3 April 2025; pp. 1035–1051. https://doi.org/10.1145/3669940.3707258.
30. Wang, X.; Zambreno, J. An FPGA Implementation of the Hestenes–Jacobi Algorithm for Singular Value Decomposition. In Proceedings of the IEEE International Parallel & Distributed Processing Symposium Workshops, Phoenix, AZ, USA, 19–23 May 2014; pp. 220–227.
31. Mur-Artal, R.; Montiel, J.M.M.; Tardós, J.D. ORB-SLAM: A Versatile and Accurate Monocular SLAM System. *IEEE Trans. Robot.* **2015**, *31*, 1147–1163.
32. Zhang, Z. A Flexible New Technique for Camera Calibration. *IEEE Trans. Pattern Anal. Mach. Intell.* **2000**, *22*, 1330–1334.
33. Triggs, B.; McLauchlan, P.; Hartley, R.; Fitzgibbon, A. Bundle Adjustment: A Modern Synthesis. In *Vision Algorithms: Theory and Practice (IWVA 1999)*; Lecture Notes in Computer Science; Springer: Berlin, Germany, 2000; Volume 1883, pp. 298–372.
34. AMD. VCU118 Evaluation Board User Guide (UG1224). AMD: Santa Clara, CA, USA, 2023. https://docs.amd.com/v/u/en-US/ug1224-vcu118-eval-bd.
35. Digilent. FMC-HDMI Reference Manual. Digilent: Pullman, WA, USA, 2014. https://digilent.com/reference/_media/fmc_hdmi:fmc-hdmi_rm.pdf.
36. AMD. Vivado Design Suite User Guide: Power Analysis and Optimization (UG907). AMD: Santa Clara, CA, USA, 2026. https://docs.amd.com/r/en-US/ug907-vivado-power-analysis-optimization.
37. OpenCV Team. OpenCV 4.10.0 Documentation. 2024. https://docs.opencv.org/4.10.0/.

Disclaimer/Publisher's Note: The statements, opinions and data contained in all publications are solely those of the individual author(s) and contributor(s) and not of MDPI and/or the editor(s). MDPI and/or the editor(s) disclaim responsibility for any injury to people or property resulting from any ideas, methods, instructions or products referred to in the content.
